# Mathematical modelling of angiogenesis using continuous cell-based models

**DOI:** 10.1007/s10237-016-0784-3

**Published:** 2016-04-01

**Authors:** F. D. Bookholt, H. N. Monsuur, S. Gibbs, F. J. Vermolen

**Affiliations:** 1 Delft Institute of Applied Mathematics, Delft University of Technology, Delft, The Netherlands; 2 Department of Dermatology (VUmc), VU University Medical Center, MOVE Research Institute Amsterdam, Amsterdam, The Netherlands; 3 Department of Oral Cell Biology, Academic Centre for Dentistry Amsterdam (ACTA), University of Amsterdam and VU University Amsterdam, MOVE Research Institute Amsterdam, Amsterdam, The Netherlands

**Keywords:** Finite-element method, Cell-based model, Angiogenesis, In vitro experiments, Endothelial cells, Stalk cells, Tip cells

## Abstract

In this work, we develop a mathematical formalism based on a 3D in vitro model that is used to simulate the early stages of angiogenesis. The model treats cells as individual entities that are migrating as a result of chemotaxis and durotaxis. The phenotypes used here are endothelial cells that can be distinguished into stalk and tip (leading) cells. The model takes into account the dynamic interaction and interchange between both phenotypes. Next to the cells, the model takes into account several proteins such as vascular endothelial growth factor, delta-like ligand 4, urokinase plasminogen activator and matrix metalloproteinase, which are computed through the solution of a system of reaction–diffusion equations. The method used in the present study is classified into the hybrid approaches. The present study, implemented in three spatial dimensions, demonstrates the feasibility of the approach that is qualitatively confirmed by experimental results.

## Introduction

Angiogenesis is the process through which a new blood vessel is formed from a pre-existing blood vessel network. An adequate blood vessel network is required to supply blood to the entire human or animal body. In cases of (mechanical) damage, like a wound, the small blood vessel network in the wounded area has been disrupted and needs to be restored. In other cases of damage, one can think of the re-establishment of a vascular network around cardiac (coronal) arteries that may have closed as a result of atherosclerosis. In the aforementioned processes, angiogenesis is indispensable for the survival of the organism. During the early stages of development of a tumour, growth occurs through cell division and proliferation. Subsequently, it halts as a result of lack of oxygen and even develops a necrotic core. Finally, it is able to continue growing if a vascular network around the tumour has been developed. Here, angiogenesis is responsible for turning a benign tumour into a malignant tumour, which will possibly metastasise (or spread out) to other parts of the body, often leading to morbid and mortal consequences for the patient.

In order to understand the underlying mechanisms of angiogenesis, it is important to carry out experiments both in in vitro and in in vivo settings. Since qualitative (images) and quantitative (after analysis) results are obtained from these experiments, it is important to quantify and to test the hypotheses that are formed after theoretical assessment and analysis of the results. Therefore, mathematical modelling of phenomena like wound healing, wound contraction, tumour growth, ulcer development and angiogenesis has become very important and has developed into a mature state. The maturity of the modelling can be seen from the number of approaches that are used to simulate the aforementioned processes. Some of the approaches treat cells by the use of averaged quantities where the models end up as continuum-scale partial differential equations for cell densities. Examples of such continuum-scale approaches in the context of wound healing are Britton and Chaplain (1993), Javierre et al. (2009), Prokharau et al. (2014), Valero et al. (2014) and Gaffney et al. (2002), Maggelakis (2003), Maggelakis (2004) in the context of angiogenesis. The list of examples is far from complete. Next to the continuum-scale approaches, several formalisms have been developed on the smaller cell colony scale, where we start with mentioning the relevant work in Oers et al. (2014), Graner and Glazier (1992), Merks and Koolwijk (2009) on cellular Potts modelling in the context of angiogenesis. The cellular Potts models fall within the class of cellular automata models, which divide the computational domain into a discrete set of lattice points. Each lattice point is either occupied or not occupied by a cell (or by one of the subdomains) based on several biologically derived constrained optimality principles. Since cells or their boundaries move in a discrete fashion, and since intra-cellular adherence can be built in easily as a penalisation, the use of cellular Potts models has become a very natural choice for the simulation of angiogenesis where endothelial cells move and stay attached to each other. On the same cell colony scale, we mention the semi-continuous approach, where cells are treated as discrete entities, but where their migration is not restricted by any lattice points. Here, several modelling approaches have been developed in the context of wound closure, wound contraction, cell migration, and tumour growth and development. Examples of such models are the studies in Byrne and Drasdo (2009), Groh and Louis (2010), Mousavi et al. (2013), Neilson et al. (2011), Rey and Garcia-Aznar (2013), Vermolen and Gefen (2012), Vermolen and Gefen (2015), Vermolen et al. (2015). A recent review on particle methods applied to wound healing and tumour growth can be found in Vermolen (2015). Regarding cancer initiation, growth and invasion of cancer cells, and the use of cell-based modelling, we refer to the studies by Schlüter et al. (2014) and Vermolen et al. (2015). One can distinguish between models in which the cell geometry does not change over the simulation and those models where cells actually geometrically deform. An example of 3D models where cells deform and migrate, using a probabilistic voxel finite-element method, is given in Borau et al. (2014). This voxel finite-element is stochastic, as well as discrete, whereas some cell deformation models are based on phase-field methods or on moving surface partial differential equations like in, respectively, the work by Marth and Voigt (2014) and Elliott et al. (2012).

Looking at the process of angiogenesis, one finds the classical continuum-scale models along with the cellular automata approaches; however, hybrid approaches combining cell-based approaches with finite-element simulations are very scarce in the literature. Regarding bone growth and angiogenesis, we refer to the work of our Belgian colleagues (Carlier et al. 2012) where endothelial tip cells are moving individually in a lattice-free manner and where other cells are treated in terms of cell densities. Chemotactic and haptotactic signals determine the migration of tip cells. Several chemotactic factors as growth factors are taken into account by approximating the solution of a system of diffusion–reaction equations. This interesting work also treats impaired angiogenesis in a framework with two spatial dimensions, where angiogenesis is considered in the context of bone formation. Another interesting study on modelling angiogenesis where mechanical cues were taken into account was done by Stéfanou et al. (2015). The present approach that we consider in this study deals with the modelling of an experimental in vitro setting, where a fibrin matrix is considered with a confluent monolayer of endothelial cells on top submerged in an extracellular fluid. Our approach is three dimensional where all endothelial cells are treated as soft spheres. Further, we distinguish between tip and stalk cells, and opposed to Carlier et al. (2012); stalk cells and tip cells are able to differentiate to either (sub-)phenotypes at all times, see Tammela et al. (2011), Tung et al. (2012) and Blanco and Gerhardt (2014) for an experimental justification. The concentrations of the growth factors are treated analogously to the work in Carlier et al. (2012) in terms of diffusion–reaction equations. However, we also include proteins that are secreted by the tip cells through mathematical point sources which make the stalk cells follow them based on the mechanism of chemo/haptotaxis. Next, to chemotaxis, a durotaxis term is added such that the cells preferably stay near the transition of the extracellular fluid and fibrin matrix (hence approximately at the transition from fluid to matrix, that is, approximately at the basement membrane). To the best of our knowledge, we believe that these additions are innovative and complementary to the existing literature.

**Fig. 1:**
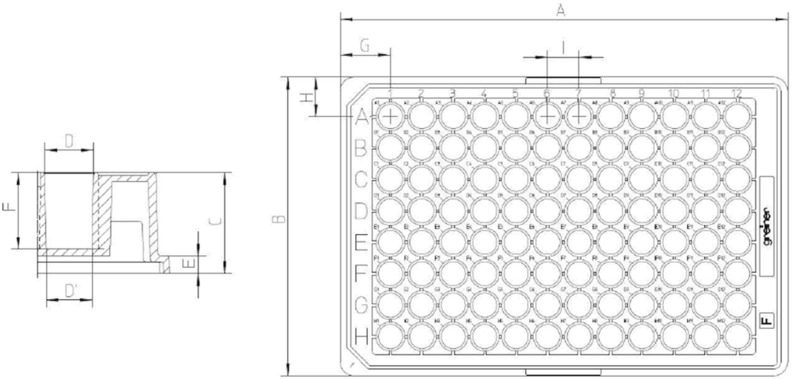
Standard 96-well plate. Wells are cylindrical with a diameter of 7 mm and a total volume of around 300 $\upmu$ L

The paper is organised as follows: in Sect. 2, the experimental set-up is presented, and subsequently in Sect. 3, the mathematical formalism and the numerical solution strategy are presented. This description is followed by the presentation of the simulation results in Sect. 4. Finally, in Sect. 5, the model is discussed and some conclusions are drawn.

## The experimental set-up

**Fig. 2:**
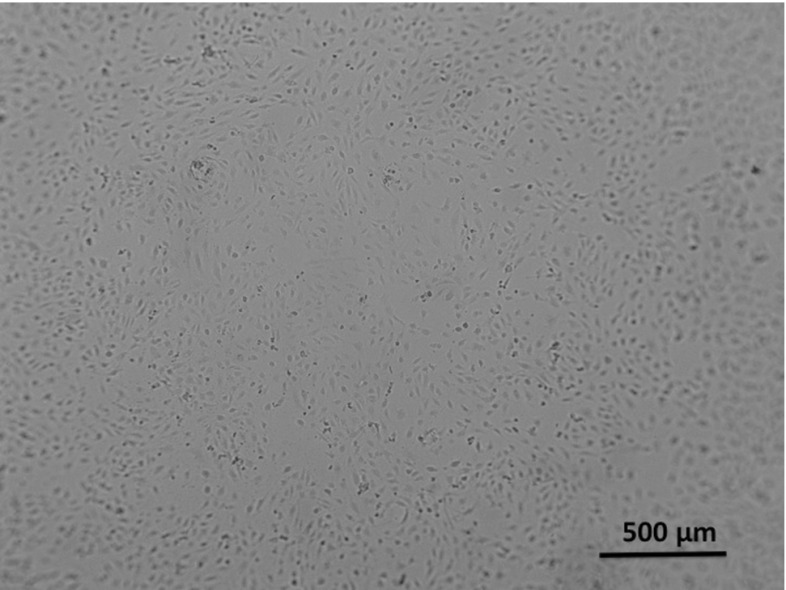
Dermal ECs in a control well. No sprouting can be seen

The dermatology department of the VUmc carries out several in vitro assays using primary Human Dermal Tissue Endothelial Cells (DTECs) on different substrates like fibrin. In this study, our particular focus is on the sprouting assay which uses a fibrin matrix and which is carried out in a standard 96-well plate depicted in Fig. 1, see Koolwijk et al. (1996) for the development of the assay. In this assay, angiogenic responses to the angiogenic growth factor vascular endothelial growth factor (VEGF) are measured for different concentrations. On the first day, a $v_F = 100\,\upmu$L fibrin matrix ($3\frac{\mathrm{mg}}{\mathrm{mL}}$ fibrinogen with $0.5 \frac{\upmu \mathrm{g}}{\mathrm{mL}}$ thrombine IIa) is placed in a total of 39 wells on top of which a 100 $\upmu$L solution is poured containing around $N = 20.000$ ECs. The total volume in the well then is $v = 200\,\upmu$L. Experimental observations show that ECs have a typical diameter of around $45\,\upmu$m, and hence, a radius of $R = 22.5\,\upmu$m. ECs are ellipsoidal being twice as long as wide. The ECs sink and adhere to the fibrin matrix, thus forming a confluent monolayer covering the surface of the fibrin matrix as depicted in the microscopic images in Fig. 2.

On the second day, the cells are stimulated using different conditions. Three wells serve as baseline controls, where no growth factors are added. All other wells are treated with 2 $\frac{\mathrm{ng}}{\mathrm{mL}}$ TNF-$\alpha$ to maintain and activate the monolayer of EC. In addition, most wells are treated with additional growth factor VEGF in different concentrations. All different concentrations are replicated in threefold to compare the results, and the well numbers are used to label the microscopic images. We summarise the different concentrations in Table 1.

**Table 1:** Control wells have nothing added to them

C	T	VT 1.1	VT 3.3	VT 10	VT 25
C	T	VT 1.1	VT 3.3	VT 10	VT 25
C	T	VT 1.1	VT 3.3	VT 10	VT 25

All wells with a “T” have a $2 \frac{\mathrm{ng}}{\mathrm{mL}} = 2 \times 10^{-3} \frac{\upmu \mathrm{g}}{\mathrm{mL}}$ (microgram per millilitre) TNF-$\alpha$ solution added. Wells with a “V” have VEGF added to them in the given concentrations in mg/mL. The numbers behind “VT” stand for the amount VEGF in the unit of mg/mL added to these wells

**Fig. 3:**
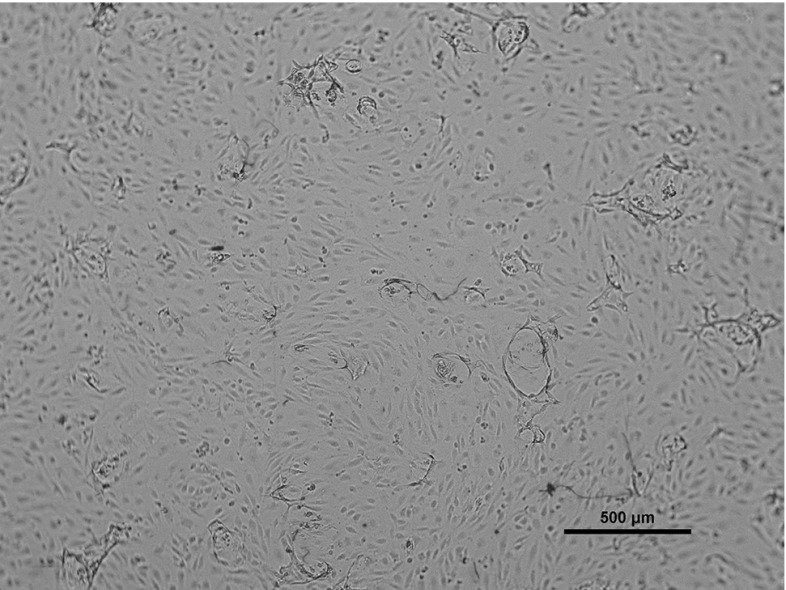
Dermal ECs in a control well after stimulation with 25 ng/mL VEGF and 2 ng/mL TNF-$\alpha$. The circular structures form the boundaries of newly formed sprouts

**Fig. 4:**
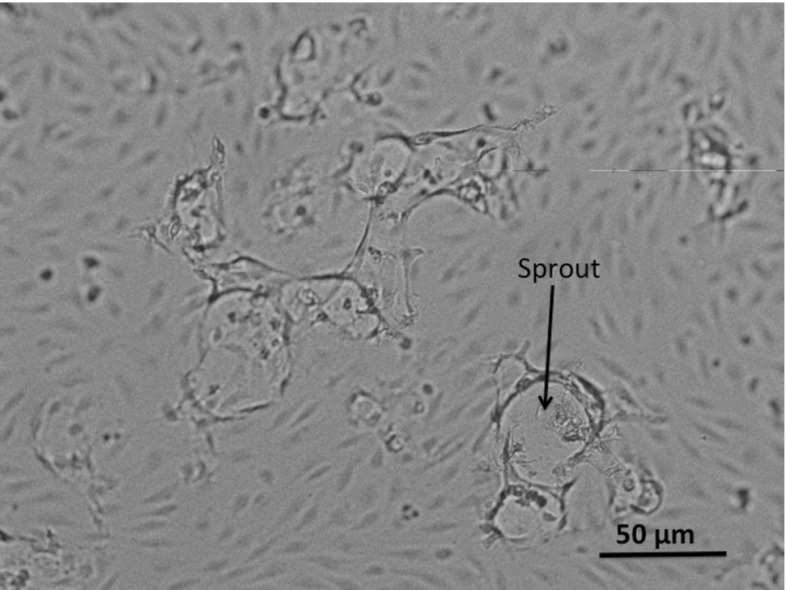
Dermal ECs in a well after stimulation with 25 ng/mL VEGF and 2 ng/mL TNF-$\alpha$. The circular structures form the boundaries of newly formed sprouts. This figure represents a magnification of ten times with respect to Fig. 3. One of the sprouts has been indicated by an *arrow*

Depending on the donor-specific endothelial cell motility, fibrin matrices are fixated 48–72 h after stimulation. The sprouting into the fibrin matrix is observed using microscopic images like those in Fig. 3. In this figure, we see cells stimulated with VT25. The monolayer is roughly undamaged, except for a couple of circular-like structures with dark edges. These dark edges form the premises of the newly formed sprouts and are most likely the effect of the fibrous layer underneath the monolayer bending out of the focal reach of the microscope. In Fig. 4, we zoom in on one of the sprouts, where one of the sprouts has been indicated by an arrow. Inside the sprout, the fibrin matrix is degraded and this shows up slightly lighter on the microscopic image. We can see that no ECs show up in the image inside the sprout. This is due to the fact that the sprouts move into the matrix and get out of focus in the microscopic image. The amount of sprouting in an assay is quantified using image processing software. The darker edges of the sprouts are coloured, and the cumulative area of the coloured regions is calculated as a percentage of the total area of the image. This percentage will be called *P*(*t*), and this variable will serve as a measure of sprouting. Although it cannot be seen in these microscopic pictures, we know that sprouts are, as a rule of thumb, twice as deep as their diameter at the top of the fibrin matrix. One can conclude this by varying the focal depth of the microscope. Sprouts usually are in downwards direction, but slightly bending sprouts are also observed.

### Driving forces on cells in sprouting angiogenesis

The motility of cells on the fibrin matrix is subject to many mechanical and biological factors. We identify several factors driving the movement of cells on the fibrin matrix. The mathematical formulation of these principles is covered in Section 3. For detailed cell biological descriptions of the hereafter listed phenomena, we refer the reader to the extensive work on cell movements by Bray (2001).

#### Chemotaxis

Gamba et al. (2003) and Serini et al. (2003) describe chemotaxis as the movement of cells in response to a chemical stimulus. One speaks of positive (negative) chemotaxis if the movement is in the (opposite) direction of the gradient and the chemical is called a chemoattractant (chemorepellent). Chemoattractants can be, following the Keller–Segel model formulated by Horstmann (2003), secreted by the cells themselves, leading to the formation of isolated clusters of cells. The chemotactic process takes place thanks to pseudopodia on the cell membranes that are formed on the sides of the cell in high concentrations of the chemoattractant and “reach” towards higher concentrations, pulling the cell in the desired direction. Inflammatory mediators such as TNF-$\alpha$ may increase the motility of cells.

#### Cell–cell forces, contact mechanics

Cells can adhere to each other by physically attaching their cell membranes using surface proteins like cadherins. ECs adhere to each other using vascular endothelial cadherin (VE-cadherin) bonds. VE-cadherin at the same time works as an inhibitor of haptotactic movement caused by VEGF by binding to the same receptor used in the chemotaxis signalling pathway. Merks describes this contact inhibition in his cellular Potts Model in Merks and Koolwijk (2009). ECs have a certain optimal elliptical shape induced by their cytoskeleton and will try to elastically return to this shape upon deformation. The magnitude of these forces is proportional to the elasticity of the cell and the severity of the deformation. This deformation can be caused by cells colliding into one another. We will denote this effect by contact mechanics in further chapters.

#### Cell–matrix forces, durotaxis

Transmembrane integrin proteins on the cell membrane adhere to fibrous scaffolds such as fibrin matrix or collagen and exert contractile forces causing cell–matrix adhesion. Since these forces are caused by physical attachment to the fibrin matrix, the net force will be in the direction of the fibrin matrix gradient. However, high-density fibrin matrix may be too stiff for the cells to move into. The same cell–matrix adhesive forces cause strain in the elastic fibrin matrix, which on its turn is sensed by other cells adhering to the matrix, and they get pulled along the stress lines. This effect is called mechanotaxis. Reinhart-King et al. (2008) conducted a series of experiments considering the interplay between cell–cell adhesion and mechanotactic forces for endothelial cells. They conclude that matrix stiffness is an important factor for the cell motility and the ability to mechanically communicate through the substrate.

## The mathematical model

First the model formulation is presented, and this is followed by the presentation of the numerical method.

### The mathematical formulation

**Fig. 5:**
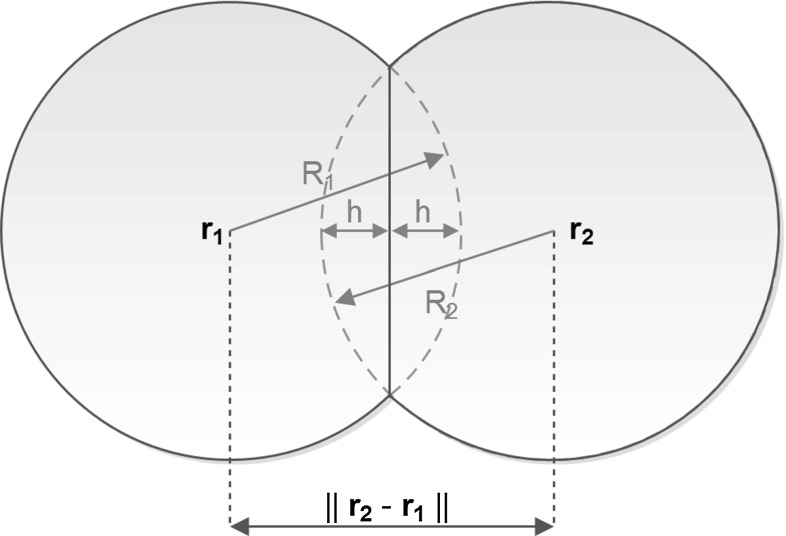
A schematic of the chemical interaction between the tip and stalk cells in which the tip cells secrete the the DLL4 to make the stalk cells follow them. The tip cells start migrating as a result of the gradient of the VEGF. Further, the arrangement of the fibrin matrix, basement membrane and extracellular fluid is shown, as well as the degradation of the basement membrane and fibrin matrix by, respectively, the chemicals MMP and uPA. In the model itself, gravity is not dealt with; however, in the simulations, the cells are seeded on the top surface of the basement membrane by first positioning them on top of the extracellular fluid and let them “sink” (by gravity) onto the top surface of the basement membrane to get a somewhat more randomised arrangement of endothelial cells as initial configuration for the simulations. In the sprouting assay set-up used at the VUmc, gravity together with contact mechanics forms a reasonable explanation for the formation of the initial confluent mono-layer. This initial configuration is also determined by the contact forces that the cells experience when seeded on the top of the basement membrane

In this section, we present the governing equations with their boundary and initial conditions. We consider a three-dimensional cylindrical domain $\varOmega$ with boundary $\partial \varOmega$. Initially the domain is divided into three segments: $\varOmega _E$, $\varOmega _B$ and $\varOmega _F$, denoting the regions occupied by the, respectively, from top to bottom, extracellular fluid, basement membrane and fibrin matrix, see Fig. 5 for a sketch. The basement membrane can be considered as a somewhat stiffer top layer of the fibrin matrix. Since the tip cells will chemically create holes through the boundary membrane and fibrin matrix by degrading fibrin, the extracellular fluid will occupy the channels formed by the tip cells. To this extent, the biological problem could be considered as a moving boundary problem. This approach will not be used, and in the approach that we propose, we introduce the volume fractions of fibrin matrix, basement membrane and extracellular fluid, which are, respectively, denoted by $f_F(t,\mathbf{x})$, $f_B(t,\mathbf{x})$ and $f_E(t,\mathbf{x})$, where $\mathbf{x} = (x,y,z)$, being the coordinates of the location. Hence initially, we set

1 $$\begin{aligned} f_p(0,\mathbf{x}) = {\left\{ \begin{array}{ll} 1, &{}\quad \mathbf{x} \in \varOmega _p \\ \\ 0, &{}\quad \mathbf{x} \notin \varOmega _p, \end{array}\right. } \quad \text {for }\; p \in \{E,B,F\}. \end{aligned}$$

Since the basement membrane and fibrin matrix are similar collagen-structured materials and since the extracellular fluid is a fluid, we introduce the variable $f_S(t,\mathbf{x}) := f_B(t,\mathbf{x}) + f_F(t,\mathbf{x})$ being the solid fraction. In our mixture formulation, we require that $f_S(t,\mathbf{x}) + f_E(t,\mathbf{x}) = 1$ at all times *t* and at locations in $\varOmega$.

**Fig. 6:**
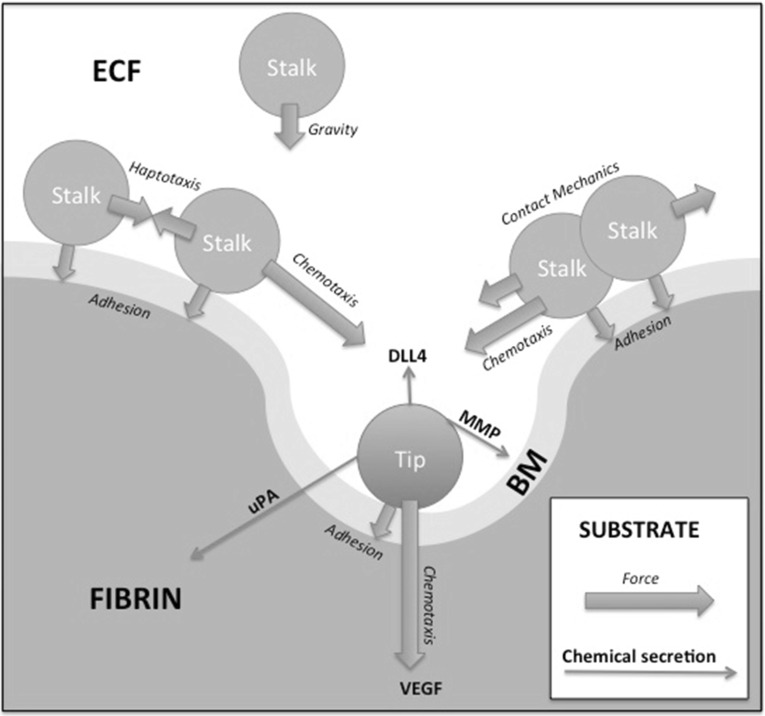
A schematic of the contact forces caused by partial overlapping of two spherical cells. *Note* that the picture displays a two-dimensional representation, whereas the implementation is in three spatial dimensions

#### Cell dynamics

The endothelial cells are treated as discrete spheres with radius *R*. This simplification has been chosen to facilitate a non-complicated approach for the intercellular contact forces. In case of ellipsoid cells, then, contact mechanics would need the determination of the points and angles of contact to compute the resulting direction of cellular displacement. The treatment of the contact forces in the case that cells are colliding against each other has been illustrated in Fig. 6, where the cells are considered to have collided if $h > 0$. In the case that two cells collide, the contact force is directed in the direction of the line connecting the centres of the cells. In case of multiple cells that collide, then the net contact force is obtained from a linear combination from all contributions from the separate cells. More information regarding this topic can be found in Vermolen and Gefen (2013). We distinguish between tip (leading) and stalk endothelial cells, see Fig. 5. The only phenotype we consider here is endothelial cells. The spatial positions of the cells are denoted by $\mathbf{x}_i(t)$, where *i* and *t*, respectively, denote the cell index and time. To distinguish between the tip cells and stalk cells, we introduce the set of all the stalk cells,

2 $$\begin{aligned} S(t) := \{i \in \{1,\ldots ,n\}~:~ \text {cell } i \text { is a stalk cell} \}, \end{aligned}$$

and its complement

3 $$\begin{aligned} T(t) = S^c(t) = \{i \in \{1,\ldots ,n\}~:~\text {cell } i \text { is a tip cell} \}. \end{aligned}$$

Note that the transitions of the cells between the two states “stalk” and “tip” make the sets time dependent. It is assumed that the gradient of the vascular endothelial growth factor concentration, $c_V(t,\mathbf{x})$, drives the chemotactic movement of the tip cells. Next to the chemotactic signal, we take durotaxis into account where we realise that the stiffness of the material is proportional to the fraction of the volume of solid, see Griffith (1921) where experimental results confirming that solid is stronger than liquids. We take durotaxis into account by considering the gradient of $f_S(t,\mathbf{x})$. We also postulate that the cells are not willing to move into a very dense solid. To this extent, we want the cells to move towards the centre of the solid–liquid interface, denoted by $\Gamma (t)$, which we define implicitly by the surface

4 $$\begin{aligned} \Gamma (t) = \left\{ \mathbf{x} \in \varOmega ~:~f_S(t,\mathbf{x}) = \frac{1}{2} \right\} . \end{aligned}$$

We interpret the cell’s willingness to reside at locations near $\Gamma (t)$ as their adherence to the fluid–solid interface. To this extent, the tip cells move according to

5 $$\begin{aligned} \hbox {d} \mathbf{x}_i(t)= & {} \left( \alpha M(\mathbf{x}_i(t)) \mathbf{z}_i + \gamma \nabla c_V(t,\mathbf{x}_i(t))\right. \nonumber \\&\left. +\, \lambda (f_S(t,\mathbf{x}_i(t))) \nabla f_S(t,\mathbf{x}_i(t))\right) \hbox {d}t \nonumber \\&+\, \sqrt{2 D} \hbox {d} \mathbf{W}(t), \qquad i \in T(t). \end{aligned}$$

Here, $\mathbf{x}_i(t)$ denotes the cell’s centre position at time *t*. The first term of the right-hand side in the above equation denotes the component of migration as a result of contact forces between neighbouring cells as well as forces that cells exert on the substrate, which are sensed by the other cells if the strain energy is large enough. Here *M* denotes the strain energy density and $\mathbf{z}_i$ denotes the direction of movement determined by the strain energy density. The variable $\alpha$ models the mobility of cell, as well as its viability and the friction forces applied onto the cell surface as it moves over the solid material inside the channel.

This term has been detailed in Vermolen and Gefen (2012), but for this study we implemented the following changes with respect to Vermolen and Gefen (2012):

- The formulation has been extended to three-dimensional geometry, as in Dudaie et al. (2015);
- The formulation involves regions with different structures, and in the extracellular fluid, there is no long-distance communication that is associated with long-distance mechanical signals;
- The cells are moving around in a non-homogeneous lattice where the elasticity modulus changes over time and location. This issue is currently dealt with by averaging the elasticity modulus used to determine how the strain energy density signal attenuates over the line between the two cells;
- The friction has been adjusted to incorporate the various solid surfaces, as well as to model the difficulty cells experience in $\mathbb {R}^3$ to move through cavities, the friction coefficient $\tilde{\mu }$ in Vermolen and Gefen (2012) has been modified to
  6 $$\begin{aligned} \mu (\mathbf{x}_i) = \frac{\tilde{\mu }}{E_F} \sum _{k \in \{F,B,E\}} f_k(\mathbf{x}_i) E_k, \end{aligned}$$
  where $E_k$ stands for the elasticity modulus in each phase.

Therewith the strain energy density associated with long-distance communication is written as

7 $$\begin{aligned} \tilde{M}(\mathbf{x}_i) = M_i^0 + \sum _{j \ne i} M_{ij}, \end{aligned}$$

where

8 $$\begin{aligned} M_i^0 = \frac{F_i^2 (1 - f_E)}{32 \pi ^2 E_S(\mathbf{x}_i) R^4}, \end{aligned}$$

in which $F_i$ represents a mechanical force exerted by viable cells, which we treat as a constant in the present study. Further, *R* denotes the radius of the cells. The local elasticity modulus, $E_S(\mathbf{x}_i)$, is determined by a mixing rule, which reads as

9 $$\begin{aligned} E_S(\mathbf{x}) = \frac{E_B f_B + E_F f_F}{f_B+f_F}. \end{aligned}$$

Note that as $f_E \rightarrow 1$, that is $f_B, f_F \rightarrow 0$, then $M_i^0 \rightarrow 0$, which models that if the cells are not able to adhere to a solid, then they will not generate any force, and thus, no strain energy density is generated. Further, $F_i$ denotes the cellular traction force exerted by cell *i*. The attenuation of the signal over the domain surrounding cell *j* towards cell *i* is modelled by

10 $$\begin{aligned} M_{ij} = \frac{F_i^2 (1 - f_E)}{32 \pi ^2 E_S(\mathbf{x}_i) R^4} \cdot \exp \left( -\frac{\overline{E}_S(\mathbf{x}_i,\mathbf{x}_j)}{E_C} || \mathbf{x}_j - \mathbf{x}_i || \right) ,\nonumber \\ \end{aligned}$$

where $\overline{E}_S(\mathbf{x}_i,\mathbf{x}_j)$ represents the averaged elasticity modulus between the two communicating cells, computed by

11 $$\begin{aligned} \overline{E}_S(\mathbf{x}_i,\mathbf{x}_j) = \frac{E_S(\mathbf{x}_i) + E_S(\mathbf{x}_j)}{2}. \end{aligned}$$

Further, $E_C$ represents the elasticity modulus of the cell. Mechanical contact between cells is also adjusted to the three-dimensional case using Hertz contact mechanics for two spheres, which gives

12 $$\begin{aligned}&M_{ij}^{*} = \frac{\sqrt{2}}{5 \pi } E_C\left( \frac{h_{ij}}{R} \right) ^{\frac{5}{2}}, \nonumber \\&\text {where } h_{ij} = \max (0,\frac{2R-|| \mathbf{x}_i - \mathbf{x}_j ||}{2}), \end{aligned}$$

where we refer to Fig. 6 for a schematic. Finally, the overall strain energy density is computed through

13 $$\begin{aligned} {M(\mathbf{x}_i) = \tilde{M}(\mathbf{x}_i) - \sum _{j \in \mathbb {N}_i(t)} M_{ij}^*,} \end{aligned}$$

where the index set $\mathbb {N}_i(t)$ is defined by

14 $$\begin{aligned} \mathbb {N}_i(t) := \{j \in \{1,\ldots ,n\} ~ : ~ h_{ij} > 0\}. \end{aligned}$$

The determination of the translation unit vector $\mathbf{z}_i$ is analogous to Vermolen and Gefen (2012), except for the above-mentioned adaptations.

The second term in Eq. (5) mimics the contribution as a result of chemotaxis in the direction of VEGF. Here, the concentration of VEGF is denoted by $c_V$. The $\gamma$-function incorporates the mobility of a cell, as well as the resistance by the material it has to move through, and the force that the cell is able to exert on the material. To get a dimensionally consistent relation, see Bookholt (2015) for the details, we assert

15 $$\begin{aligned} \gamma = \gamma (\mathbf{x}_i) = \frac{\beta F_i}{\rho (\mathbf{x}_i) E_S(\mathbf{x}_i)} f_S(\mathbf{x}_i), \end{aligned}$$

where

16 $$\begin{aligned} \rho (\mathbf{x}_i) = \sum _{k \in \{F,B,E\}} f_k(\mathbf{x}_i) \rho _k. \end{aligned}$$

Here $\rho _k$ are the densities of the separate phases fibrin matrix, basement membrane and extracellular fluid. As for the mechanical component, there is no chemotactic movement $f_S = 0$, that is if the cell is in an environment entirely filled with extracellular fluid.

The third term in Eq. (5) takes into account the migration as a result of durotaxis. The $\lambda$-function, $\lambda ~:~\mathbb {N} \times \mathbb {R}^+\rightarrow \mathbb {R}$, is constructed such that it is zero (no durotactic movement) in the extracellular liquid (that is $f_S = 0$), in hard solid (that is $f_S = 1$) and on the the interface $\Gamma (t)$ (that is $f_S = \frac{1}{2}$), to this extent and after some normalisation and taking into account the hypotheses that migration is inversely proportional to local stiffness, cell motility and cell viability. In Vermolen and Gefen (2012), cell viability is directly coupled to the forces that they exert, and since the dimension of $\lambda$ should be $\hbox {m}^2\,\hbox {s}^{-1}$, we use

17 $$\begin{aligned} \lambda (i,t)= & {} \frac{4^3}{3} {\hat{\lambda }} \cdot \frac{\beta _i F_i}{E_S(\mathbf{x}_i)(t)} \cdot f_S(t,\mathbf{x}_i(t))\nonumber \\&\times \left( f_S(t,\mathbf{x}_i(t))- \frac{1}{2}\right) (f_S(t,\mathbf{x}_i(t)) - 1), \end{aligned}$$

where $\beta _i$ denotes the motility coefficient of cell *i* and where $\hat{\lambda }$ denotes the adhesive scaling factor, which is treated as a constant. The last term of the right-hand side of Eq. (5) models random walk of the cells (diffusion), where *D* denotes the diffusion coefficient of the endothelial cells. The vector $\hbox {d} \mathbf{W}(t)$ has three entries that are all independent normally distributed events with zero mean and a variance of $\hbox {d}t$, that is, the entries of $\hbox {d} \mathbf{W}(t)$ are Wiener Processes $\hbox {d}W_k \sim \mathcal {N}(0,\hbox {d}t)$, for $k \in \{1,2,3\}$. The tip cells secrete the protein delta-like ligand 4 (DLL4), which is the chemotactic signal of the stalk cells. Hence, for the stalk cells, with centre location $\mathbf{x}_i$, we have

18 $$\begin{aligned} \hbox {d} \mathbf{x}_i(t)= & {} \left( \alpha M(\mathbf{x}_i(t)) \mathbf{z}_i + \gamma \nabla c_D(t,\mathbf{x}_i(t)) \right. \nonumber \\&\left. +\,\lambda (f_S(t,\mathbf{x}_i(t))) \nabla f_S(t,\mathbf{x}_i(t))\right) \hbox {d}t \nonumber \\&+\, \sqrt{2 D} \hbox {d} \mathbf{W}(t), \quad i \in S(t). \end{aligned}$$

Here no distinction has been made whether cells are stalk or tip cells, since they are both endothelial cells, except for the chemotaxis term, which is driven by the gradient of the concentration of DLL4, denoted by $c_D$, where DLL4 is secreted by the tip cells. Hence by the secretion of DLL4, the tip cells make the stalk cells follow them, see Fig. 5 for a sketch of the mechanism. The stalk cells can become tip cells and, vice versa, the tip cells may become stalk cells. The transitions between these states are modelled as memoryless stochastic processes, which are classified as follows: Let $\mathcal {P}(t > \tau )$ denote the probability that the transition does not take place until time $\tau$ and let $\mathcal {P}(t > \theta + \tau ~|~ t > \theta )$ denote the probability that the transition does not take place until time $\tau + \theta$, given that the observer is at time $\theta$ where the transition has not yet taken place (that is the event did not take place before time $\theta$), then the memoryless property is defined by

19 $$\begin{aligned} \begin{array}{ll} \text {The random process is memoryless in times } t > 0\quad \\ \quad \text { if and only if } \\ \\ \mathcal {P}(t > \theta + \tau ~|~ t > \theta ) = \mathcal {P}( t > \tau ),\quad \text {for }\; \theta ,~\tau \ge 0. \end{array} \end{aligned}$$

The probability that the stalk cells become tip cells is modelled by an exponential distribution, given by the following probability density function for $t > \theta$:

20 $$\begin{aligned} f_{\pi }(i \in T(t)~|~i \in S(\theta )) = \lambda _{S \rightarrow T} \hbox {e}^{-\lambda _{S \rightarrow T} (t - \theta )}, \end{aligned}$$

for reverse transition, we analogously have

21 $$\begin{aligned} f_{\pi }(i \in S(t)~|~i \in T(\theta )) = \lambda _{T \rightarrow S} \hbox {e}^{-\lambda _{T \rightarrow S} (t - \theta )}. \end{aligned}$$

With these probability density functions, we get the following transition probabilities for $t > \theta$

22 $$\begin{aligned}&\mathcal {P}(i \in P(t)~|~ i \in Q(\theta ))\nonumber \\&\quad = \int _{\theta }^t f_{\pi }(i \in P(s)~|~i \in Q(\theta )) \hbox {d}s=1-\hbox {e}^{-\lambda _{Q \rightarrow P}(t-\theta )}, \nonumber \\&\quad \text {where } (P(s),Q(s)) \in \{S(s),T(s)\}\nonumber \\&\quad \times \{S(s),T(s)\}, \text {and } P(t)=P(s) \ne Q(\theta ). \end{aligned}$$

In the above equation, $\lambda _{Q \rightarrow P}$ is a probability rate constant. In the experimental case with large number of cells in many experimental experimental samplings, one could measure the amounts of tip cells and stalk cells, and from these figures, one can estimate the probabilities that a cell is either in the “tip state” (that is $i \in T(t)$) or in the “stalk state” (that is $i \in S(t)$). To this extent, Bayes’ theorem applied to the long-time observations gives

23 $$\begin{aligned} \frac{\mathcal {P}(i \in T(t)~|~i \in S(0))}{\mathcal {P}(i \in S(t)~|~i \in T(0))} = \frac{\mathcal {P}(i \in T(t))}{\mathcal {P}(i \in S(t))} \approx \frac{n_T(t)}{n - n_T(t)},\nonumber \\ \end{aligned}$$

where *n* and $n_T(t)$, respectively, denote the total number of endothelial cells and the number of tip cells at time *t*. The above relation gives an estimate of how the probability rates $\lambda _{S \rightarrow T}$ and $\lambda _{T \rightarrow S}$ are related. In the simulations that we will show, the probability rates depend on the chemical environment in which transitions between the two states are favoured if the VEGF concentration is high and if the DDL4 concentration is low. Some phenomenological relations have been used in this study. We finally note that the present modelling does not incorporate cell death or cell proliferation. In “Appendix”, the reader will find more details regarding the input values used in this study.

#### The proteins involved

Next we treat the concentrations of the various proteins VEGF ($c_V$), DLL4 ($c_D$), matrix metalloproteinase (MMP) ($c_M$) and urokinase plasminogen activator (uPA) ($c_U$). Note that the VEGF makes the tip cells move and further the DLL4 is secreted by the tip cells, and this chemokine makes the stalk cells follow the tip cells. In the equations, we disregard shrinkage or expansion of the total computational domain that could possibly occur due to mixing processes. All concentrations are modelled by diffusion–reaction processes, where we have for $t > 0$

24 $$\begin{aligned}&\frac{\partial c_V}{\partial t} - \nabla \cdot (D_V(f_F,f_B,f_E) \nabla c_V)\nonumber \\&\quad = -\sum _{j \in T(t)} r_V ~ c_V ~ \delta (\mathbf{x} - \mathbf{x}_j(t)), \text {in } \varOmega \end{aligned}$$

where $r_V$ is a decay rate constant due to consumption by tip cells, $D_V$ is the diffusivity of VEGF depending on the phase (fibrin matrix, basement membrane or extracellular fluid), and $\delta (.)$ represents the Dirac delta distribution, which is defined by

25 $$\begin{aligned} \begin{array}{ll} \delta (\mathbf{x}) = 0, &{} \mathbf{x} \ne \mathbf{0}, \\ \\ \displaystyle {\int _{\varOmega \ni \mathbf{0}} \delta (\mathbf{x}) d \varOmega = 1,} &{} \text {where } \varOmega \text { is open.} \end{array} \end{aligned}$$

For all the chemokines, there is flux normal (perpendicular) to the boundary, hence for $t > 0$

26 $$\begin{aligned} D_k(f_F,f_B,f_E) \frac{\partial c_k}{\partial n} = 0, \text {on } \partial \varOmega ,~ \text {for } k \in \{V,D,M,U\}.\nonumber \\ \end{aligned}$$

The initial condition for the VEGF concentration is given by

27 $$\begin{aligned} c_V(0,\mathbf{x}) = {\left\{ \begin{array}{ll} c_V^0, &{}\quad \mathbf{x} \in \varOmega _F, \\ \\ 0, &{}\quad \mathbf{x} \notin \varOmega _F. \end{array}\right. } \end{aligned}$$

DLL4 is regenerated from conversion of VEGF by the tip cells, and it is consumed by the stalk cells, to this extent, we have for $t > 0$

28 $$\begin{aligned}&\frac{\partial c_D}{\partial t} - \nabla \cdot (D_D(f_F,f_B,f_E) \nabla c_D)\nonumber \\&\quad = -\sum _{j \in S(t)} r_D ~ c_D ~ \delta (\mathbf{x} - \mathbf{x}_j(t)) \nonumber \\&\quad +\, \sum _{j \in T(t)} s_D ~ c_V ~ \delta (\mathbf{x} - \mathbf{x}_j(t)), \text {in } \varOmega . \end{aligned}$$

Here $r_D$ and $s_D$ are regeneration and consumption rates. Initially, there is assumed to be no DLL4 in the domain of computation. The metalloprotease MMP is secreted by the tip cells by conversion from VEGF, and this chemical breaks down the basement membrane, for $t > 0$, and we have

29 $$\begin{aligned}&\frac{\partial c_M}{\partial t} - \nabla \cdot (D_M(f_F,f_B,f_E) \nabla c_M)\nonumber \\&\quad = -r_M ~ c_M ~ f_B\nonumber \\&\qquad +\, \sum _{j \in T(t)} s_M ~ c_V ~ \delta (\mathbf{x} - \mathbf{x}_j(t)), \text {in } \varOmega , \end{aligned}$$

where the first term in the right-hand side models the decay of the MMP concentration as a result of the breakdown of the basement membrane, which is a somewhat stiffer extension of the fibrin matrix. This breakdown enables the Dermal ECs to migrate into the fibrin matrix (including the basement membrane). Further, $r_M$ and $s_M$, respectively, are rate constants for decay and regeneration of MMPs. Initially, the concentration of MMPs is zero at all locations of the computational domain. Finally, the protein uPA breaks down the fibrin matrix. This protein is also secreted by the tip cells, and hence, we have for $t > 0$

30 $$\begin{aligned}&\frac{\partial c_U}{\partial t} - \nabla \cdot (D_U(f_F,f_B,f_E) \nabla c_M)\nonumber \\&\quad = -r_U ~ c_U ~ f_F\nonumber \\&\quad +\, \sum _{j \in T(t)} s_U ~ c_V ~ \delta (\mathbf{x} - \mathbf{x}_j(t)), \text {in } \varOmega , \end{aligned}$$

also here the first term of the right-hand side models decay of uPA due to the breakdown of fibrin matrix. Furthermore, $r_U$ and $s_U$, respectively, are decay and regeneration rate constants regarding uPA. Initially, there is no uPA in $\varOmega$. The diffusivities are modelled using a mixing rule:

31 $$\begin{aligned} D_p(f_F,f_B,f_E)= & {} D_p^0 \left( f_F D^F \right. \nonumber \\&\left. +\, f_B D^B + f_E D^E \right) , \end{aligned}$$

where $D_p^0$ denotes the diffusivity of protein $p \in \{V,D,M,U\}$ and $D^k$ for $k \in \{F,B,E \}$ (fibrin matrix, basement membrane, extracellular fluid) denotes the diffusion factor corrected for the phase that is considered.

Since the proteins MMP and uPA, respectively, change the basement membrane and fibrin matrix into extracellular fluid, we have for $t > 0$

32 $$\begin{aligned} \left\{ \begin{array}{ll} \displaystyle {\frac{\partial f_B}{\partial t} = -r_B ~ c_M ~ f_B,} \\ \\ \displaystyle {\frac{\partial f_F}{\partial t} = -r_F ~ c_U ~ f_F, } \\ \\ \displaystyle {\frac{\partial f_E}{\partial t} = r_B ~ c_M ~ f_B + r_F ~ c_U ~ f_F.} \\ \\ \end{array} \right. \mathbf{x} \in \varOmega . \end{aligned}$$

The above relations are consistent with the requirement that the sum over all volume fractions should be equal to one.

It is noted that the above equations warrant that if there would be a sufficient number of cells that the long-time behaviour becomes $f_B,~f_F \rightarrow 0$, as well as $f_E \rightarrow 1$, along with $c_V,~c_D,~c_M,~c_U \rightarrow 0$ in $\varOmega$ as $t \rightarrow \infty$, which indicates stability of the system.

### The numerical method

To solve the stochastic differential equations for the spatial positions of the centres of the cells, the Euler–Maruyama method is used (Steele 2001). Further, the diffusion–reaction equations for the concentrations of the chemicals have been solved using the finite-element method in three spatial dimensions. To this extent, we give the weak (variational) formulation, where Sobolev/Bochner spaces are omitted, of the diffusion–reaction equation for VEGF as an example:

33 $$\begin{aligned} \left\{ \begin{array}{ll} \text {Find } c_V, \text { subject to the initial condition such that} \\ \\ \displaystyle {\int _{\varOmega } \frac{\partial c_V}{\partial t} \varphi + D(f_F,f_B,f_E) \nabla c_V \cdot \nabla \varphi d \varOmega }\\ \displaystyle {\quad = -\sum _{j \in T(t)} r_v ~ c_V(t,\mathbf{x}_j) ~ \varphi (\mathbf{x}_j), \qquad \text {for all functions } \varphi . } \end{array} \right. \end{aligned}$$

Approximating $c_V(t,\mathbf{x}) \approx \sum _{k=1}^N \overline{c}_V(t,\mathbf{x}_k) \varphi _k(\mathbf{x})$, where $\varphi _k(\mathbf{x})$ are a set of chosen basis functions (in the present study piecewise linear), and taking $\phi _i(\mathbf{x})$ for $\varphi (\mathbf{x})$, necessitates determining whether the cell centre is located in the tetrahedral element of consideration. In order to determine this, we consider a tetrahedron, *e* with vertices $\mathbf{x}_1$, $\mathbf{x}_2$, $\mathbf{x}_3$ and $\mathbf{x}_4$. We use the barycentric coordinates of the tetrahedral element. Consider tetrahedral element *e*, and let $\psi _i(\mathbf{x})$ be the linear function that is characterised by

34 $$\begin{aligned} \psi _i(\mathbf{x}_j) = \delta _{ij}, \end{aligned}$$

where $\mathbf{x}_j$ represents the vertices of *e*, and $\delta _{ij}$ denotes the Kronecker delta function. Note that $\psi _i(\mathbf{x}) = \phi _i(\mathbf{x}) \in [0,1]$ if and only if $\mathbf{x} \in \overline{e}$ and that outside the tetrahedron *e* the function $\psi$ can assume values beyond the interval [0, 1]. Further, the cell centre with coordinates $\mathbf{x}_j(t)$ is located within *e* if and only if $0 \le \psi (\mathbf{x}_j(t)) \le 1$.

An alternative treatment can be applied if the ordering of the vertices of the tetrahedral element *e* has been carried out such that the numbering over each face of the tetrahedron is in the positive orientation. Let $\varDelta$ be the determinant given by

35 $$\begin{aligned} \varDelta = det \begin{pmatrix} 1 &{} x_1 &{} y_1 &{} z_1 \\ 1 &{} x_2 &{} y_2 &{} z_2 \\ 1 &{} x_3 &{} y_3 &{} z_3 \\ 1 &{} x_4 &{} y_4 &{} z_4 \end{pmatrix}, \end{aligned}$$

then this determinant represents six times the volume of the tetrahedron since it is positive by the choice of the orientation. Further, we introduce the following auxiliary determinants that are constructed on the same principle, but now we replace the coordinates of vertex $\mathbf{x}_k$ with the coordinates of the cell centre $\mathbf{x}_j(t)$, to get $\varDelta _k$. For instance, $\varDelta _1$ is given by

36 $$\begin{aligned} \varDelta _1 = det \begin{pmatrix} 1 &{} x_j(t) &{} y_j(t) &{} z_j(t) \\ 1 &{} x_2 &{} y_2 &{} z_2 \\ 1 &{} x_3 &{} y_3 &{} z_3 \\ 1 &{} x_4 &{} y_4 &{} z_4 \end{pmatrix}, \end{aligned}$$

This should be done for all vertices of the tetrahedron *e*. If all $\varDelta _k \cdot \varDelta > 0$ for all $k \in \{1,\ldots ,4\}$, then $\mathbf{x}_k(t)$ lies within the tetrahedron *e*.

**Fig. 7:**
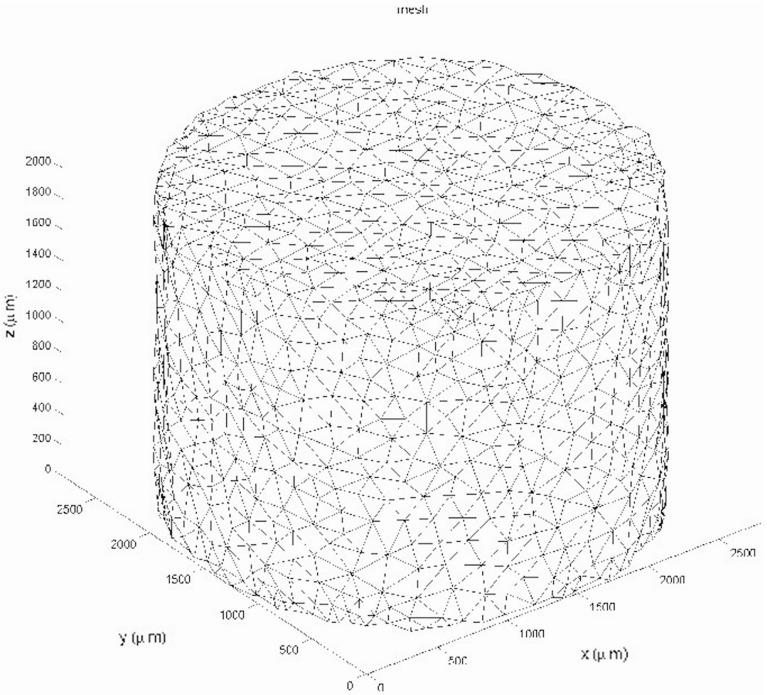
The three-dimensional finite-element mesh used in the current study. The mesh was constructed using the isomesh-mesh generator (Fang and Boas 2009)

The finite-element implementation has been done for MATLAB, where the meshes have been constructed using the iso2mesh (Fang and Boas 2009). The meshed domain is shown in Fig. 7. The iso2mesh-package generates meshes without actually seeking for an optimal bandwidth of the discretisation matrices, and to this extent, the Cuthill–McKee algorithm has been used to optimise the bandwidth. The finite-element method uses linear elements; hence, linear tetrahedra and the mass matrix needed in the time derivative are lumped through Newton–Cotes integration to prevent spurious oscillations that could even occur when implicit methods are used. Time integration of the partial differential equations is based on a first-order IMEX scheme where the diffusion operator is evaluated at the new time step, and all the nonlinear terms at the previous time step, meaning that the right-hand side and the gradient of the concentration are all treated at the new time step and that the determination of the diffusion coefficients has been performed at the previous time step. The main advantage is that the numerical stability of the time integration is not determined by the mesh size and despite this feature, one does not have to solve a complete nonlinear problem at each time step, and hence, no inner iterations are needed, and therewith, the time integration is relatively cheap. The choice of basis simple tetrahedral elements is justified because no advection terms are to be discretised, and hence, no SUPG discretisation or flux corrections are needed to suppress spurious oscillations. Higher-order finite-element methods will not improve the accuracy because of the used point sources in the partial differential equations for the concentrations (since $c_p \notin H^2(\varOmega )$ for a fixed $t > 0$, $p \in \{V,U,D,M\}$). If one aims at improving the efficiency and accuracy, then adaptive finite-element mesh strategies could be helpful, where the iso-concentration surface, implicitly defined by $f_S = 0.5$, represents the moving interface between the fluid and solid phases.

**Fig. 8:**
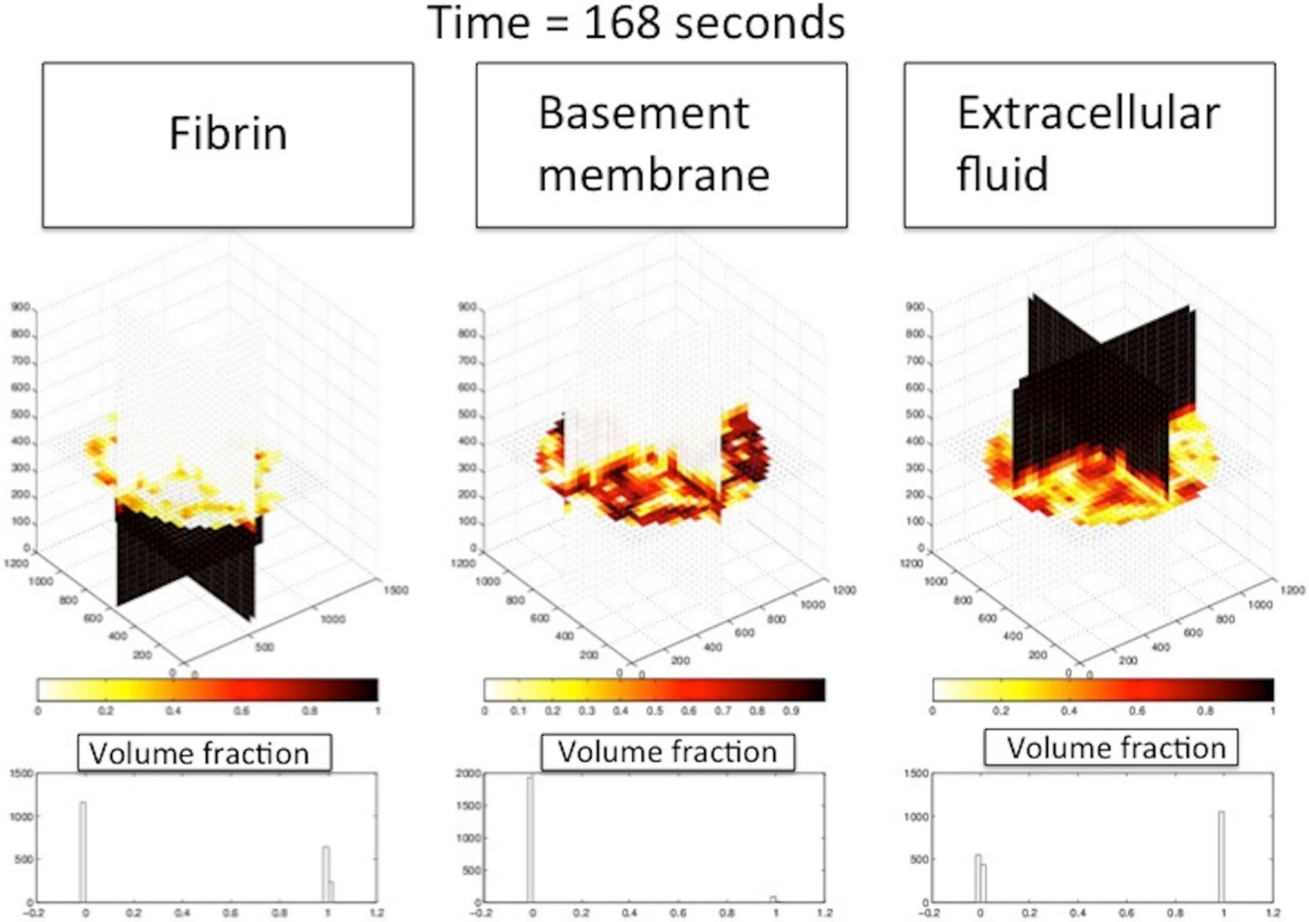
Almost initial condition plot for the substrate components using the slice plot, where the slices are perpendicular to the coordinate axes. On the *left*, *centre* and *right*, the profiles for fibrin, basement membrane and extracellular fluid are shown, respectively. *Light* and *dark colours*, respectively, represent *low* and *high* values of the volume fractions. On the *bottom*, histograms are given of the volume fractions of fibrin, basement membrane and extracellular fluid as experienced by the cells

**Fig. 9:**
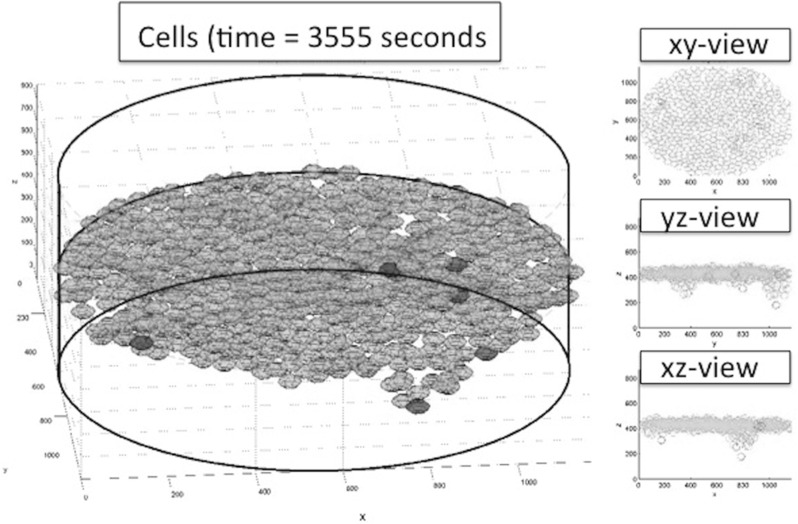
*Left* Cells plot. Tip cells are coloured *red* in the left three-dimensional plot. The *thick black lines* form the boundary of the computational domain and aid the reader in orienting the plot. The “camera” is in an angle slightly lower than the *x*, *y* plane. On the right, several projections are shown in which the tip cells are indicated by the *red crosses*

**Fig. 10:**
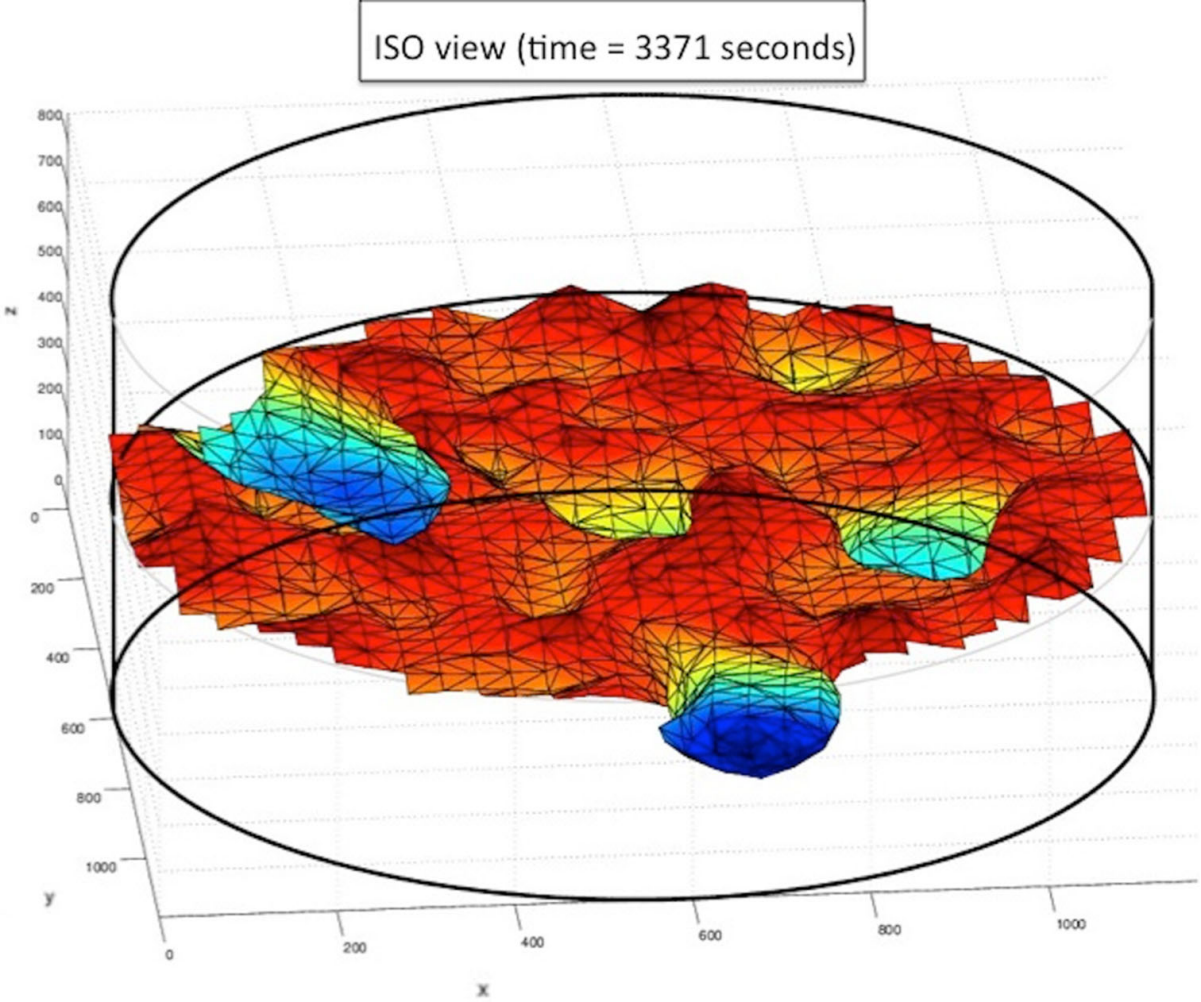
Surface plot of $F_S = 0.5$. The *thick black lines* form the boundary of the computational domain and aid the reader in orienting the plot. The “camera” is in an angle slightly lower than the *x*, *y* plane

**Fig. 11:**
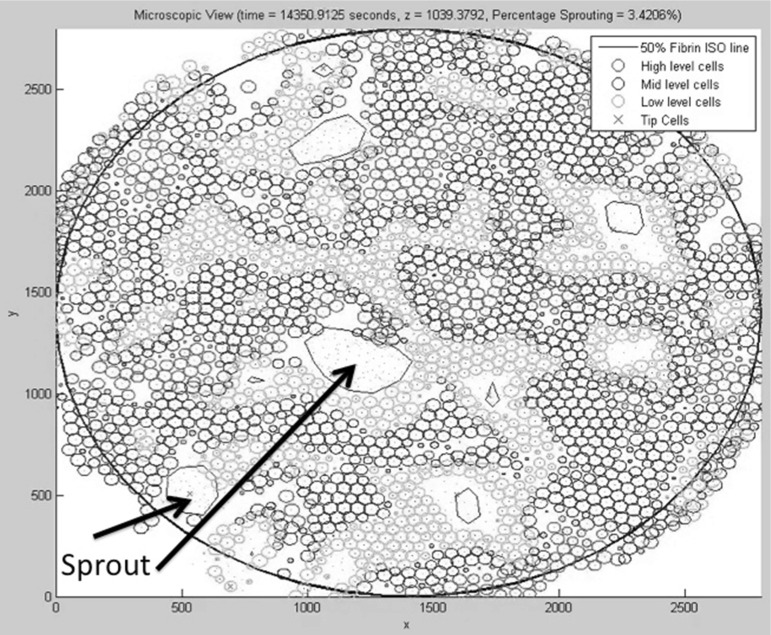
Microscopic plot of the top surface of the fibrin matrix where the cells are seeded. The *circles* represent projections of the spherical cells onto the top of the fibrin matrix. The *colours* represent cells at different heights: *Red cells* on top level, *blue cells* on middle level and *green cells* on lowest level. We see a total of $n_s = 8$ sprouts of different sizes in order of decreasing area at locations approximately $(x,y) = (1250,1200), (1100,2200), (550,550), (2200,1900), (1700,500), (1800,1000), (1000,2500)$ and (1300, 1100). We also see tip cells (denoted by *red* x-marking) that have not formed a sprout. The iso-lines are calculated in the surface at $z = 1039.4$ directly beneath the initial placement of the cells. The total number of tip cells is $n_t = 9$. Two sprouts have been indicated by *arrows*

**Fig. 12:**
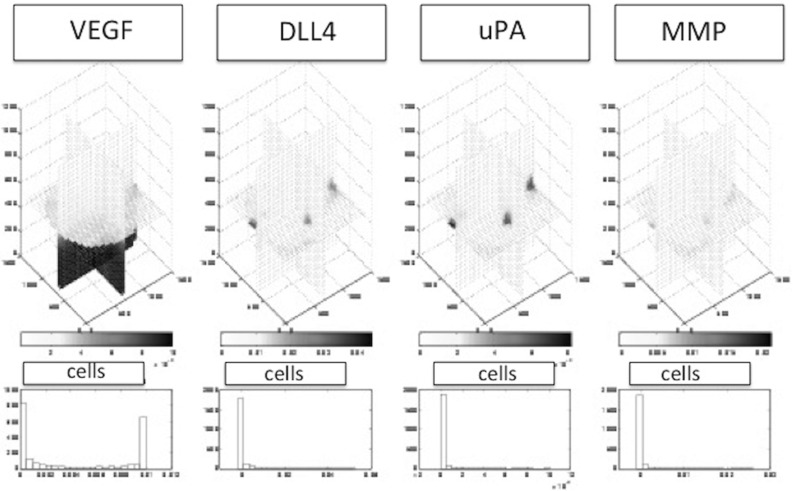
The proteins over time in slice plots following the time-dependent reaction–diffusion-sourcing equations. From left to right: VEGF, DLL4, uPA and MMP. Diffusion of the initial VEGF distribution can be observed. Furthermore, sourcing of the other three proteins at the locations of tip cells and diffusion into the surroundings can be observed. MMP and uPA react at a faster rate than DLL4 due to the abundant presence of the substrate components. At the bottom, histograms of the concentrations VEGF, DLL4, uPA and MMP (same order) are shown in terms of values experienced by the cells. This figure is taken after approximately 1 h of simulated time (time$\,=\,$3371 s)

Finally, it is noted that the numerical solution of the volume fractions is performed using Euler backward method with the concentrations that were computed earlier. One could also interchange the order: first compute the volume fractions with the use of concentrations at the previous time step and subsequently use a complete implicit Euler time integration for the numerical time integration of the concentrations. This variant has not been studied in the present study since our approach gave satisfactory results. Another alternative time-integration method is the fully coupled implicit approach, which needs an inner iteration loop within each time step. This lastmentioned approach is thought to be more expensive, and therefore, it has not been applied either.

## Simulation results

First we show the visualisation of the simulation in terms of field plots and cell plots. Subsequently, we show results in terms of quantitative measures as well as a comparison with experimental outcomes. We further carry out a sensitivity analysis on the simulations. The default input data have been listed in “Appendix”.

**Fig. 13:**
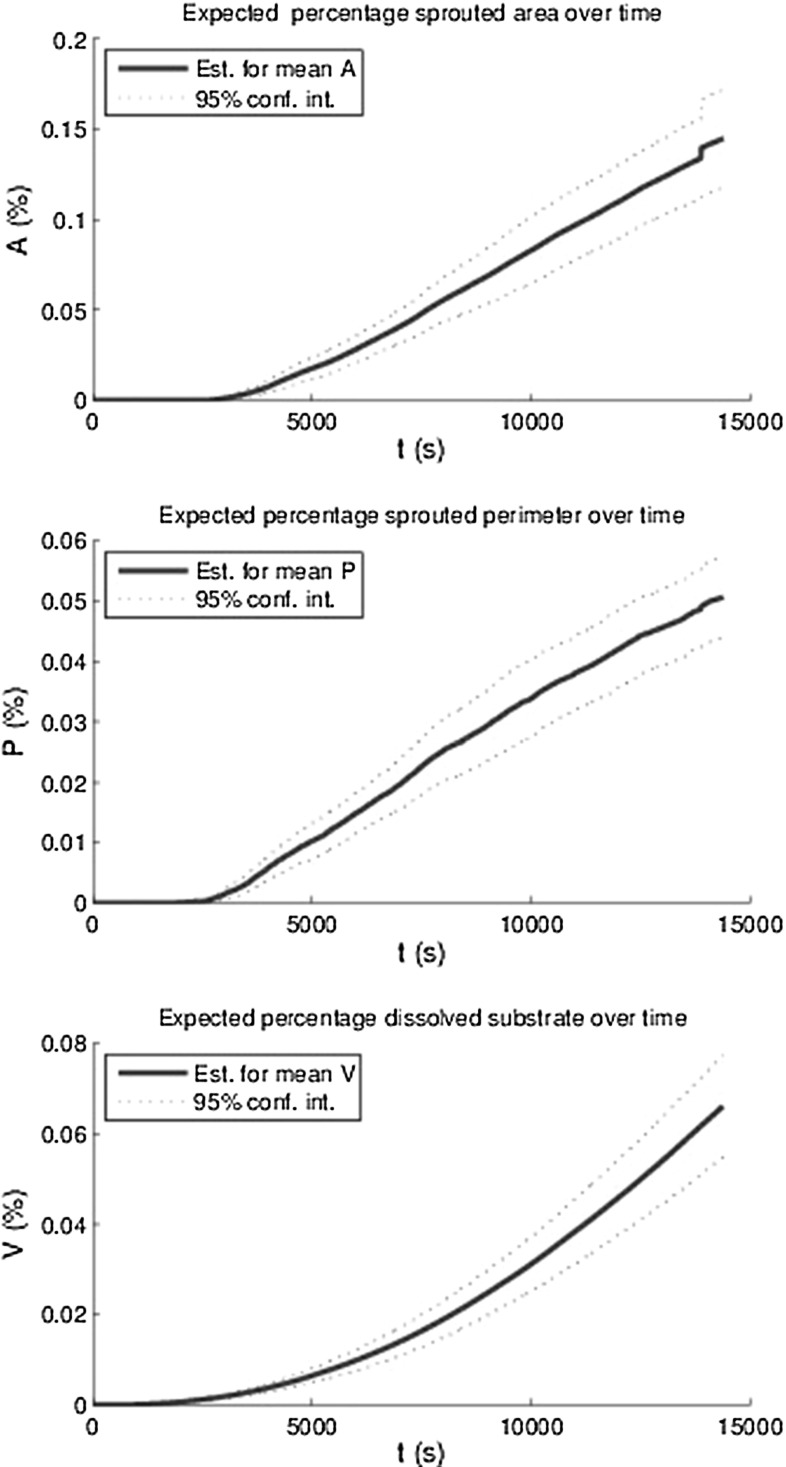
The estimators for the mean area *A*(*t*) and perimeter *P*(*t*) of all sprouts at $h_F$ as a percentage of the total area and the percentage of the total area and the percentage degraded substrate *V*(*t*) and 95 % confidence intervals based on 12 runs with identical parameters

**Fig. 14:**
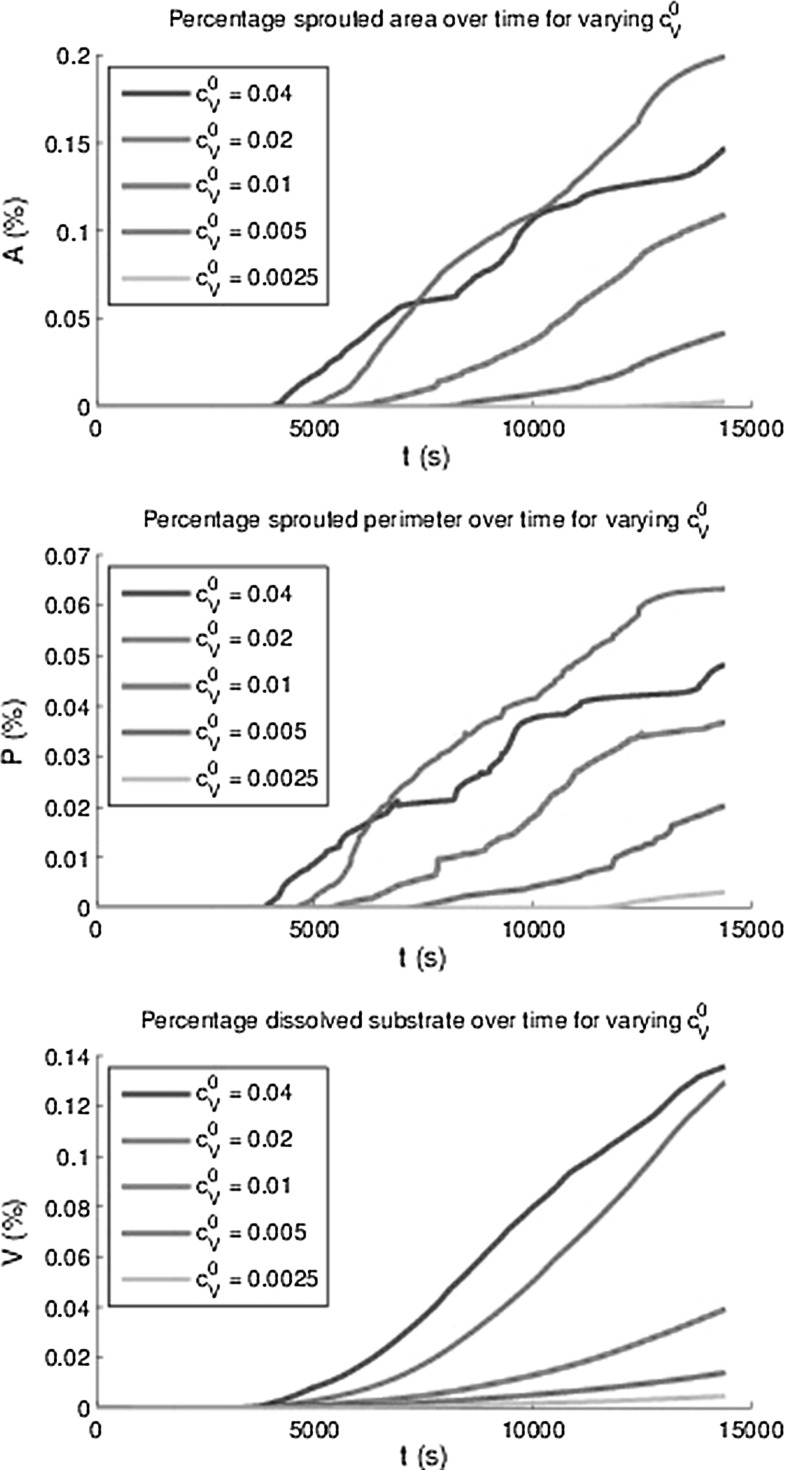
The area *A*(*t*) and perimeter *P*(*t*) of all sprouts at $h_F$ as a percentage of the total area and the percentage degraded substrate *V*(*t*) for varying initial concentrations VEGF

**Fig. 15:**
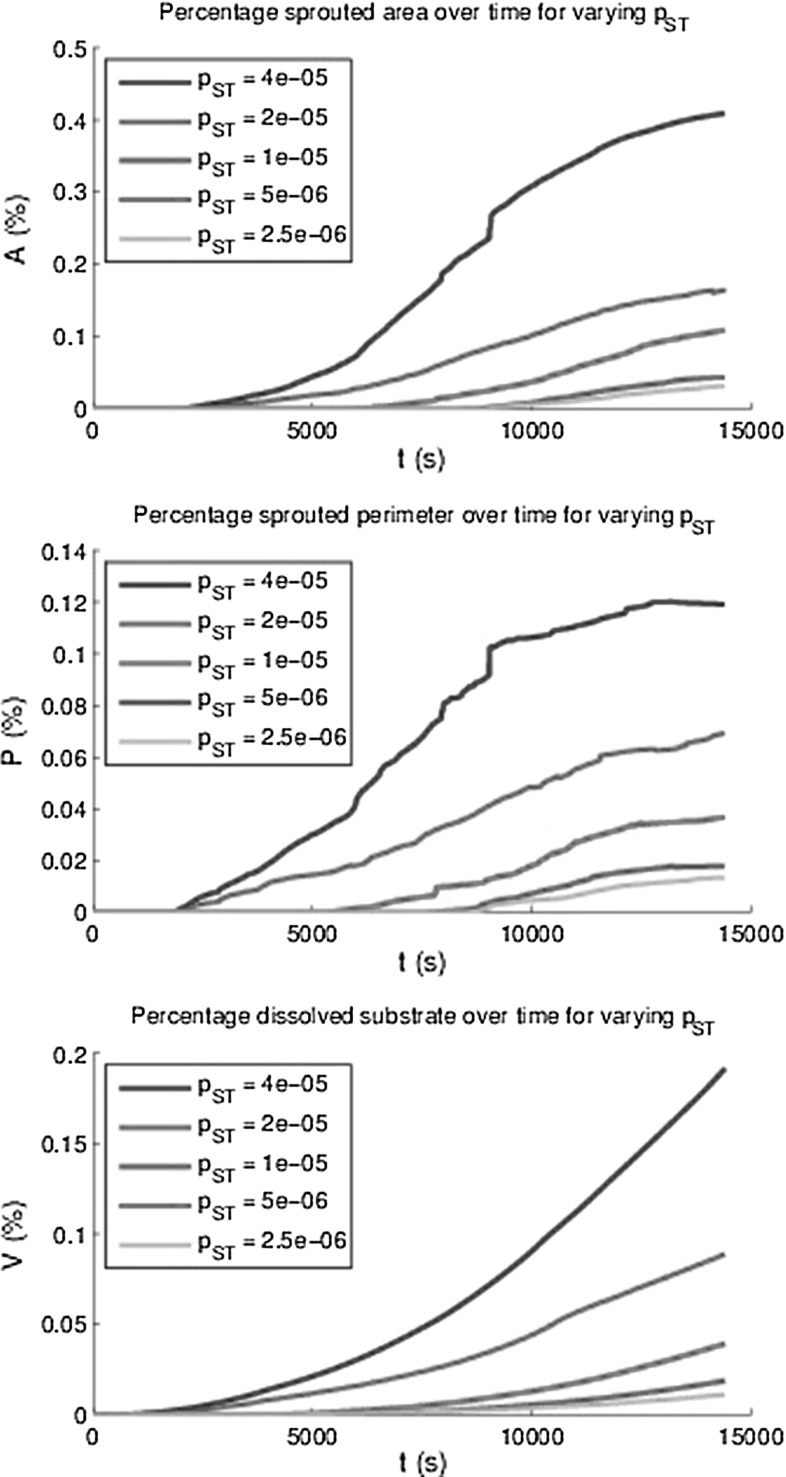
The area *A*(*t*) and perimeter *P*(*t*) of all sprouts at $h_F$ as a percentage of the total area and the percentage degraded substrate *V*(*t*) for varying maximal sprout to tip probability $\lambda _{PT}$

**Fig. 16:**
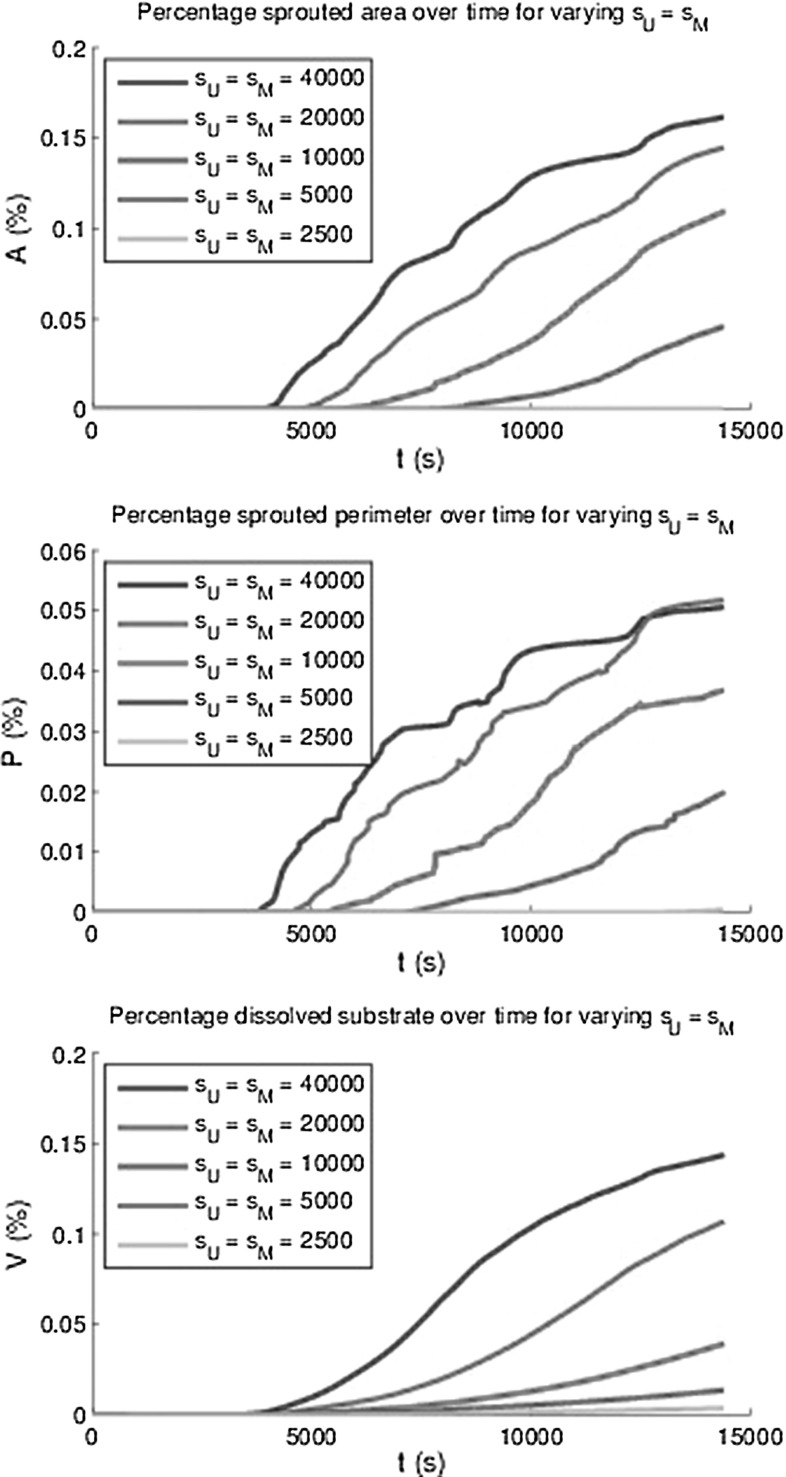
The area *A*(*t*) and perimeter *P*(*t*) of all sprouts at $h_F$ as a percentage of the total area and the percentage degraded substrate *V*(*t*) for varying MMP and uPA sourcing rates

**Fig. 17:**
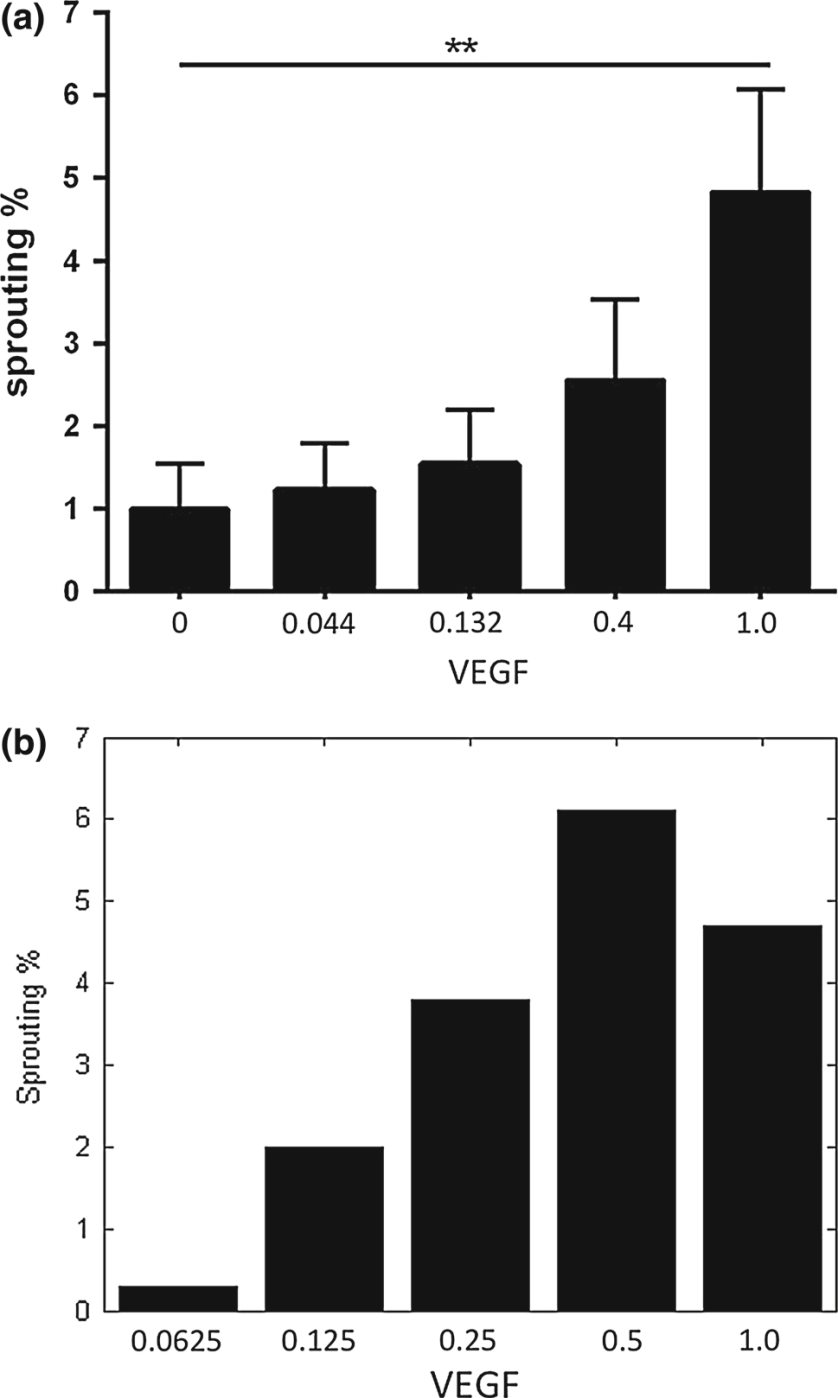
The metric *P*(*t*) as measured by the VUmc dermatology department after the third day (*left*), where the *black bars* are DTECs, and (*right*) the metric *P*(*t*) at $t = 14{,}400$ computed by the present model. The *horizontal axes* of both figures have been scaled to unity (originally the maximum of the VEGF concentrations was 25 mg/mL and 0.04 ng/$\upmu$m$^3 = 40$ mg/mL for the experiments and simulations, respectively) because of the scaling down of the chemotactic response

### Visualisation of the simulations

Initially, the cells are located on the basement membrane, and the initial volume fractions of the three phases, fibrin matrix, basement membrane and extracellular fluid are shown in Fig. 8, where the three-dimensional nature of the problem necessitates to represent the phases in a slice plot. We used slices that are perpendicular to the coordinate axes. In Fig. 8, left, the initial fibrin fraction is shown. It can be seen that the initial fibrin is distributed on the bottom. In the middle of Fig. 8, the initial fraction of basement membrane is plotted on the same slices in the domain of computation. It can be seen that the initial basement membrane is localised on a horizontal layer adjacent to the extracellular fluid and fibrin. The initial fraction of extracellular fluid is localised on positions above the membrane, as can be seen in Fig. 8 on the right. On the bottom of Fig. 8, we show the histograms of the volume fractions that are experienced by the cells. It can be seen that initially these histograms give a polarised behaviour reflecting that cells are located either in the fibrin or in the extracellular fluid to a lesser extent. The cells are modelled to migrate through chemotaxis, durotaxis (adhesion), contact mechanics and random walk. The contact mechanics prevents cells from coinciding with one another. Further, as an example, we show the positions of the cells after 3354 s in Fig. 9, where the green and red cells are the stalk and tip cells, respectively. On the left, the three-dimensional representation is shown, whereas on the right some projections are shown so that it is clearer to see how the cells fit in the channels through the fibrin matrix. It can be seen in this figure that the tip cells are localised on positions closest to the bottom since they indeed take the lead in chemically creating holes in the basement membrane and fibrin. The tip cells are also shown in the projections on the right, where they are represented by the red crosses. In Fig. 10, on the left side, the level surface of $f_S = 0.5$ is shown, which is the surface on which we let the cells adhere to. This figure shows how the channels have been formed by the cells through the release of MMP and uPA that convert the basement membrane and fibrin matrix into extracellular liquid. Hence, this iso-surface of $f_S = 0.5$ shows a time instant in the time evolution of the region that is occupied by the solid phases, and it can be used to visualise the evolution of angiogenesis if different time frames are shown after one other. Next to this figure, we show the positions of the cells that are residing on the top surface of the basement membrane, that is in the vicinity of the initial interface between the extracellular liquid and the basement membrane in Fig. 11. Like in the experimental setting, we show cells that are within a layer of three cell diameters around the initial position of the interface between the basement membrane and the extracellular fluid. The top, middle and lower layer cells have been plotted in red, blue and green, respectively, in Fig. 11. On the projections, the tip cells are represented by the red crosses. In Fig. 11, several gaps arise (see the white regions surrounded by the black lines and where we indicated two of them by arrows, see also Fig. 4 in the in vitro experiments). These gaps coincide with sprouts formed by the tip cells. We compute their areas and compare these areas that have been computed for the same setting in the in vitro experiments. The concentrations of all the chemicals are obtained by the finite-element approximation of the solution to the three-dimensional diffusion–reaction equations in which the cells either consume or regenerate the chemicals at their spatial positions through point sources or sinks. Sometimes, one observes some cells that are located within the circumferences of sprouts in Fig. 11, such as in the white patch on the top left. The probable reason for this observation is that these cells just detach from their neighbours that are still on the top surface and start migrating downwards into the sprout that is being formed. Note that here this sprout is very premature and that here the $F_S = 1/2$ level curve has not formed yet on the top layer of the solid, which means that the summed solid fractions are still above 0.5 there. Since the sprout is occupied with extracellular fluid, the migration of the cell does not proceed instantaneously, and hence, they remain visible on the top (though they are not located on the interface between the solid and fluid phases) for a while. The concentrations of the VEGF, DLL4, uPA and MMP after 3766 s are shown in Fig. 12 in terms of slice plots on the planes perpendicular to the coordinate axes, where it can be seen that the gradients are largest in the vicinity of $f_S = 1/2$ where the tip cells chemically create holes in the solid phases. On the bottom of the figure, histograms of the concentration that all the cells experience have been shown. It can be seen that at the top of the fibrin matrix, the VEGF concentration has decreased a bit as a result of consumption by the tip cells, whereas further away to the bottom, the concentration has not changed considerably. The concentration of DLL4 increases slightly in the vicinity of the top surface of the fibrin matrix since this ligand is secreted by the tip cells that are predominantly at the interface between the extracellular fluid and the solid substances. This ligand makes the stalk cells follow the tip cells. A similar behaviour is observed for the uPA and MMP concentrations, which, respectively, are responsible for the degradation of the fibrin matrix and basement membrane. Next to the concentration plots, we plot histograms of the values of all the concentrations that are experienced by the cells, which determine to what extent the stalk cells will follow the tip cells. Many cells have not moved yet and at their positions, the concentration of all proteins is between zero and very low values. The other cells that did move (tip cells and stalk cells with positions initially adjacent to the tip cells) are entering the region where VEGF has higher values. Therewith there is a considerable portion of cells that experience high values of VEGF. Furthermore, in the course of time VEGF stimulates secretion of the other proteins by tip cells that make the stalk cells follow them and that facilitate the degradation of the fibrin matrix and basement membrane.

### Quantitative measures from the simulations

In order to quantify angiogenesis, several measures have been introduced. The first measure is the total area of the sprouts on the initial top layer of the basement membrane divided by the total basement membrane area. This measure is denoted by *A*(*t*). The second measure is called the sprouted perimeter, which is obtained by computing the total perimenter of the sprouts on the top of the basement membrane multiplied by the cell diameter and subsequently divide this result by the total area of the basement membrane. We denote this measure by *P*(*t*). The third measure is the total volume fraction of the sprouts computed by the integral over $f_E$ over the initial fibrin matrix domain. The lastmentioned measure is denoted by *V*(*t*). Since the model contains a stochastic nature, through migration and differentiation, it is important to determine the amount of uncertainty for the set of parameters used. The results have been plotted in Fig. 13, where the mean curves for the respective quantities have been plotted over time as well as the 95 % interval of confidence (exceeding probability of $p=0.05$) for all these quantities using 12 runs with identical choices for all parameters. It is clear that all the measures go up as the vascularisation process continues. Further, there is an incubation time, which is a result of the following sub-processes:

- Endothelial cells become tip cells by a random selection in the model and only after a short, nonzero length period, the first tip cells appear;
- The VEGF concentration has to reach the tip cells in order to be able to degrade the basement membrane and fibrin matrix;
- The (tip) cells can only migrate quickly to the bottom provided the basement membrane and fibrin matrix have decayed.

Besides the incubation time, a small jump (at $t \approx 14000$) in the plot for the percentage of the sprouted area is observed. After having examined the video for this simulation, it turned out that two sprouts merged and that the routine to compute the total area of the sprouts counted the merged sprouts twice. This small jump is not visible in the computation of the volume fraction of the vessels. Simulations have been done with different adhesion values $\hat{\lambda }$ in the durotaxis term, and the dependence did not seem to be significant since the behaviour was not monotonic and the variations were not larger than the the variations in different runs with identical input parameters. The input values for $\hat{\lambda }$ ranged between 6 and 100. Therefore, these results are not shown here. Possibly using lower values could show more dependency but this is not certain since the adhesion is not the main trigger for the vascularisation process. Lowering the $\hat{\lambda }$-value only allows cells to move towards the $f_S = 0.5$ in a slower manner and the time-integration method would allow for less overshoot. Furthermore, the initial VEGF concentration has been varied and higher initial VEGF concentrations predict a higher volume of vascularisation, see the bottom of Fig. 14. Note that we only plot the mean of all the 12 runs that were carried out. Furthermore, the other measures were computed over time for the various initial VEGF concentrations, and the monotonic behaviour was also observed except for the highest VEGF concentrations. This deviation is attributed to the fact that higher initial concentrations give a larger concentration gradient. Since the total movement of the cells is determined by contact forces, protein signals, durotaxis (for the adherence) and random walk, the chemotactic and haptotactic components to the overall movement of the cells increases as the initial concentration increases. Thereby the path that the cells follow towards the bottom will be more according to a straight line, and hence, the cross sectional will be lower than in the case that the movement of the cell is distorted more from all the other signals such as contact forces and random walk. In Fig. 15, we show the influence of the variation of the probability that stalk cells become tip cells. It can be seen that the amount of vascularisation in all the measures increases monotonically with the probability of stalk cells to become tip cells. Finally, we show the influence of the regeneration of uPA and MMP’s in the evolution of the vascularisation over time in Fig. 16. Since both concentrations act in a similar way, we took the regeneration constants equal. It can also be seen that the vascularisation rate monotonically increases with the regeneration constants. The decrease in speed at the latest times is due to flattening out of the VEGF signal due to diffusion, which is the main trigger for the further growth of the sprouts. This is also confirmed by the absence of a dependence of the sprouting dynamics upon changing the $\hat{\lambda }$-parameter.

### Comparison with experiments

Finally, we compare the simulation results to the outcomes obtained from the experiments in terms of the metric *P*(*t*), which accounts for the perimeter over time. The results can be seen in Fig. 17, where human dermal tissue (DTECs) endothelial cells are considered. In both the in vitro and simulation experiments, the concentration of VEGF was varied and the sprout perimeter *P*(*t*) was computed after the third day in the in vitro experiments and after 14,400 s (4 h) in the simulations. This discrepancy in times was caused because the right parameter values were not yet available. Firstly, it is noted that the trends of increasing vascular perimeter are observed in both simulations for increasing values of VEGF concentration. However, in the simulations it can be seen that for the largest value of initial VEGF concentration, the amount of sprouting seems to drop. This drop is attributed to the migration mechanism of the endothelial cells: for larger values of the initial concentration in the fibrin matrix region, the magnitude of the VEGF concentration is larger. Thereby the chemotaxis movement becomes larger. Since for all the cases the other mechanisms that contribute to migration (cell–cell contact, random walk and durotaxis) are approximately the same, the relative portion of chemotaxis is larger if the initial VEGF concentration in the fibrin matrix is larger. Therefore, the paths and holes that the cells will make through the solid will be more straight with fewer migrational components perpendicular to normal of the initial interface between solid and fluid. Hence, for larger initial concentrations, the chemotaxis component dominates and the cells will migrate straight towards the bottom, by which the small vessels will have a smaller diameter and a smaller perimeter. If the concentration of VEGF would be much larger in the experimental case, then one possibly observes the same behaviour for the relation between *P* and the initial stimulation with the VEGF concentration, since results of in vitro experiments often reveal a bell-shaped curve in the response of cells to increasing concentrations of a stimulus. Probably it is a matter of adjusting the parameters to more appropriate values to have a drop in the sprouting percentage at a higher concentration. This behaviour cannot be attributed to a chemical saturation effect. Furthermore, adjusting the parameter values will also lead to a decrease of the discrepancy between the times at which we determined the sprouting percentages in Fig. 17. A more efficient implementation of the present model will be needed if one wants to match the in vitro experiments to the simulations through inverse modelling. This inverse modelling will make a better fit between simulations and the in vitro experiments possible. Since the aim of the current paper is to introduce the mathematical model which is original in its kind, being a cell-based model for angiogenesis, this inverse modelling is omitted here.

## Discussion and conclusions

In this section, we discuss the model and give various recommendations for further study, and the final conclusions are drawn.

### Discussion

We based most parameters and scaling factors in the derivation of the cell movement model on physical or biological principles. The only parameter that forms the exception is the dimensionless scaling factor $\hat{\lambda }$. This parameter mediates the contiguity of the monolayer of cells; however, in our sensitivity analysis we have seen that this parameter does not play an important role in angiogenesis. The addition of natural protein decay over time would be an interesting property to add to the system of PDEs. We have performed a sensitivity analysis on five parameters, and we did twelve model runs for the estimator of the mean of all sprouting metrics. Extra computational power or parallelisation could reduce the cost of the simulations so that these numbers can be improved. Such a parallelisation has recently been carried by Woods et al. (2014). Another interesting approach is to apply a full continuum model in terms of a system of partial differential equations like by Maggelakis (2003), Maggelakis (2004) or by Gaffney et al. (2002). The latter approach allows to consider angiogenesis on a larger scale such as on a tissue scale. It would be of great interest to apply some of the homogenisation techniques that, for instance, are currently applied in porous media applications to link the microscopic, cellular, scale to the macro, tissue scale. One could also use an optimisation in the cheaper continuum modelling approach as a start for the cell-based model. The optimisation could be refined in a cell-based formalism, which in total gives a hybrid (multi-scale) regression technique.

Obtaining appropriate values for the physical parameters is often very hard and in many cases impossible in complicated models. An example is the diffusive speed $D_V$. For most of the parameters, we used values that were reported in the literature, and if no values in literature were reported, then we used educated guesses so that the model predictions are reasonable. The difference of the VEGF concentration between the in vitro study and the initial concentrations used in the simulations was compensated for by scaling down the chemotactic response accordingly. A modification of these values will not change the model outcomes significantly. As mentioned earlier, one could use sound inverse modelling techniques to get access to modified parameter values which could reduce the gap between experimental and simulation outcomes. At this stage, we are satisfied with having developed a new sound hybrid cell-based model for angiogenesis.

In the current model, the edges of the domain do not exert any forces on the cells, making it possible for cells to move out of the computational domain. Cells that have moved out of the domain are not within an element of the FEM mesh and therefore cannot sense or source any proteins or react to the substrate properties. This fact on itself forms no problem for the rest of the computational model, but does require much useless computational effort, predominantly in trying to find the (non-existing) element a cell is located in. An improvement would be to remove these cells from the computations. Another approach could be to give the boundaries contact mechanical properties or to lay a monolayer of ghost cells on the boundaries that provide the contact mechanical forces to keep the cells in the problem domain.

In the formulation of the rates $\lambda _{S \rightarrow T}$ and $\lambda _{S \rightarrow T}$ of tip cell selection, we normalise the VEGF and DLL4 concentrations with the initial concentration VEGF $c_v^0$. In hindsight, this is no reasonable assumption since tip cell selection does not depend on the absolute VEGF concentration, but rather on a saturation with respect to the initial condition. It would be an improvement to remove this normalising factor and reconsider these rates. It would be very interesting to see whether we can verify more of our simulation results with laboratory measurements. This incorporates measurement of other metrics from the in vitro experimental results than only *P*(*t*) and maybe 3D visualisation of the sprouting assay using multi-focal plane microscopy or other techniques.

In the future, we want to combine the current angiogenesis model with modelling of cancer development, such as in Vermolen (2015), where the necrotic core of the tumour releases growth factors (Tumour Necrosis Growth factor) that trigger the angiogenesis response of the endothelial cells.

### Conclusions

Our model is qualitatively successful in describing the in vitro angiogenesis sprouting assay as performed by the VUmc dermatology department. We modelled the degrading of the substrate by proteases secreted by ECs as a continuous process dependent on the properties of the substrate itself. Cell motility is modelled using a cell-based formalism based on mechano-biological principles that are well established in cell biology. A probabilistic model based on local chemical conditions is proposed to model the differentiation of ECs into tip cells and stalk cells.

The proposed metrics of the *amount of sprouting* seem to align with the in vitro results on a qualitative level. Quantitative comparison is hard due to many uncertainties, both in the proposed computational model and in the measurement techniques used for the in vitro experiment. The morphology of the sprouts is similar to the experimental setting.

The metrics over time produced by the model respond to variation in parameters as we would expect from biological reasoning. Only the variation of the VEGF concentration is performed in the laboratory setting, and the results are comparable. The area of sprouted perimeter ranges from 1 to 5 % in the in vitro experiments and ranges from 0.5 to 6 % in the simulations for varying concentrations VEGF, which are of the same order of magnitude.

VEGF concentrations, protease secretion rates and the probabilistic model for tip cell selection are important factors in sprout formation process. We postulate that it is the tip cell’s ability to degrade the substrate in its surroundings that drives the success of producing a viable sprout as well as give rise to the sprouts proliferation speed and its final depth. This factor is at least as important as the chemotactic response to a higher concentration VEGF or the adhesive properties. The success rate of sprout formation for a tip cell is between 50 and 60 %, independent of the number of tip cells present. We postulate that also in vitro the number of tip cells is larger than the number of sprouts.

The model was constructed to simulate angiogenesis. Vasculogenesis is another process witnessed in studies containing ECs as described by Nany et al. (2004) and Merks et al. (2004). Since our formalism describes EC behaviour in a general sense, we also witness vasculogenesis-like structure formation for varying values of the substrate elasticity. Since modelling vasculogenesis was not the scope of this study, we leave further investigation of this phenomenon for future research.

## Appendix: Input data

ECs can change from stalk cells into tip cells (and reversibly) based on the local protein balance. Higher concentrations of VEGF will increase the chance of a stalk cell to become a tip cell. However, a nearby tip cell that sources DLL4 inhibits a stalk cells from becoming tip cells.

We model the changing time of a stalk cell to become a tip cell using an exponential distribution with a rate $\tilde{\lambda }_{ST}$. We also model the changing time of a tip cell to change back into a stalk cell using an exponential distribution with rate $\tilde{\lambda }_{TS}$. The probability density function (PDF) of the exponential distribution is given by

$$\begin{aligned} f(t,\lambda ) = {\left\{ \begin{array}{ll} \lambda \hbox {e}^{-\lambda t} &{}\quad \text {for } t \ge 0,\\ 0 &{}\quad \text {for } t < 0. \end{array}\right. } \end{aligned}$$

The changing times are then distributed according to exponential distributions. The rates $\tilde{\lambda }_{ST}$ and $\tilde{\lambda }_{TS}$ are dependent on cell-specific factors and local chemical conditions. Since the concentration VEGF $c_V$ only decreases over time, we know that we have $c_V^{\max } = c_V^0$ and we normalise using $\frac{c_V}{c_V^{\max }}$. The concentration DLL4 $c_D$ can take arbitrary positive values but in practice almost never exceeds the concentrations VEGF so again we “normalise” by setting $\frac{c_D}{c_V^{\max }}$. For ideal circumstances, i.e. $c_V = c_V^{\max }$ and $c_D = 0$, we define the maximal rate of changing from stalk to tip per second to be $p_{ST}$. The rate $\tilde{\lambda }_{ST}$ increases for increasing $c_V$ and decreases for increasing $c_D$. We model

37 $$\begin{aligned} \tilde{\lambda }_{ST}(c_V, c_D) = p_{ST} \hbox {e}^{-p_{s}\left( 1-\frac{c_V}{c_V^{\max }}\right) } \hbox {e}^{-p_{i}\frac{c_D}{c_V^{\max }}}. \end{aligned}$$

The strength of the DLL4 induced inhibition is governed by the parameter $p_i$ (*i* for inhibition) and higher values of $p_i$ constitute stronger inhibition. The strength of the stimulation by VEGF is governed by the parameter $p_s$ (*s* for stimulation), and lower values of $p_s$ constitute stronger stimulation even for low values of $c_V$. Tip cells can change back into stalk cells at a rate governed by the decreasing of a VEGF gradient. We model

38 $$\begin{aligned} \tilde{\lambda }_{TS}(c_V) = p_{TS} \hbox {e}^{-p_s\frac{c_V}{c_V^{{\max }}}}, \end{aligned}$$

and set $p_{TS} = 1 \times 10^{-6}$. The rates in Eqs. 37 and 38 are per second. During a time step of length $\Delta t$, we see that we have the following probability of a cell type change, where we approximate the exponential with its first-order Taylor expansion around $t = 0$.

$$\begin{aligned} \int _0^{\Delta t} \tilde{\lambda } \hbox {e}^{-\tilde{\lambda } t} dt= & {} \left[ 1 - \hbox {e}^{-\tilde{\lambda }t }\right] _0^{\Delta t} = 1 - \hbox {e}^{\tilde{\lambda } \Delta t}\\&\approx 1 - (1 - \tilde{\lambda }\Delta t) = \tilde{\lambda }\Delta t. \end{aligned}$$

This approximation is suitable for small enough time steps $\Delta t$, and we have to specifically ensure that $\tilde{\lambda }_{TS}\Delta t \le p_{TS}\Delta t \le 1$. Bayes theorem now dictates that $\frac{P(S|T)}{P(T|S)} = \frac{P(S)}{P(T)}$ and we end up with an equilibrium ratio between tip cells and stalk cells given the local chemical conditions.

**Table 2:** Domain parameters

Parameter	Symbol	Code name	Value	Dimension	Source
Domain scaling factor	$\xi$	g.dom.scaling	[1–10]	–	–
Diameter well	$d_\mathrm{top}$	g.dom.dTop	7000	$\upmu \hbox {m}$	Experiment
Volume well	*v*	g.dom.vol	200	$\upmu \hbox {L}$	Experiment
Volume fibrin matrix	$v_F$	g.dom.volFibrin	100	$\upmu \hbox {L}$	Experiment

**Table 3:** Chemical simulation parameters

Parameter	Symbol	Code name	Value	Dimension	Source
Substrate threshold	$f_0$	g.chemP.minThres	Boolean	–	–
Diffusion coef. VEGF	$D_V^0$	g.chemP.dV	1.00	$\upmu \hbox {m}^2\,\hbox {s}^{-1}$	Plank et al. (2002)
Diffusion coef. DLL4	$D_D^0$	g.chemP.dD	0.51	$\upmu \hbox {m}^2\,\hbox {s}^{-1}$	Est. on Plank et al. (2002)
Diffusion coef. uPA	$D_U^0$	g.chemP.dU	1.23	$\upmu \hbox {m}^2\,\hbox {s}^{-1}$	Est. on Plank et al. (2002)
Diffusion coef. MMP	$D_M^0$	g.chemP.dM	0.53	$\upmu \hbox {m}^2\,\hbox {s}^{-1}$	Est. on Plank et al. (2002)
Diffusion factor Fibrin matrix	$D^F$	g.chemP.dF	1.00	–	–
Diffusion factor BM	$D^B$	g.chemP.dB	2.00	–	–
Diffusion factor ECF	$D^E$	g.chemP.dE	0.10	–	–
Reactive rate VEGF	$r_V$	g.chemP.rV	0.024	$\upmu \hbox {m}^3\,\hbox {s}^{-1}$	Plank et al. (2002)
Reactive rate DLL4	$r_D$	g.chemP.rD	0.024	$\upmu \hbox {m}^3\,\hbox {s}^{-1}$	Est. on Plank et al. (2002)
Reactive rate. uPA	$r_U$	g.chemP.rU	0.024	$\hbox {s}^{-1}$	Est. on Plank et al. (2002)
Reactive rate MMP	$r_M$	g.chemP.rM	0.024	$\hbox {s}^{-1}$	Est. on Plank et al. (2002)
Reactive rate Fibrin matrix	$r_F$	g.chemP.rU	1.210	$\upmu \hbox {m}^3 \hbox {ng}^{-1}\,\hbox {s}^{-1}$	Lutolf et al. (2003)
Reactive rate BM	$r_B$	g.chemP.rM	1.210	$\upmu \hbox {m}^3 \hbox {ng}^{-1}\, \hbox {s}^{-1}$	Est. on Lutolf et al. (2003)
Sourcing rate DLL4	$s_D$	g.chemP.sD	10.00	$\upmu \hbox {m}^3 \hbox {s}^{-1}$	–
Sourcing rate uPA	$s_U$	g.chemP.sU	10.00	$\upmu \hbox {m}^3 \hbox {s}^{-1}$	–
Sourcing rate MMP	$s_M$	g.chemP.sM	10.00	$\upmu \hbox {m}^3 \hbox {s}^{-1}$	–
Initial density VEGF	$c_V^0$	g.chemP.iV	0.01	$\hbox {ng} \upmu \hbox {m}^{-3}$	–

**Table 4:** Cell parameters

Parameter	Symbol	Code name	Value	Dimension	Source
The number of cells in the well	*N*	g.cell.nCells	20.000	–	Experiment
Radius of an EC	*R*	g.cell.rCell	22.5	$\upmu \hbox {m}$	–
Elastic modulus of an EC	$E_c$	g.cell.Ec	10	$\hbox {nN}\upmu \hbox {m}^{-2}$	Kuznetsova et al. (2007)
Maximal exerted force of an EC	$F_i$	g.cell.Fi	1000	nN	Reinhart-King et al. (2003), Ganz et al. (2006)
Motility of the cell surface	$\beta _i$	g.cell.beta	0.02	$\hbox {s}^{-1}$	Vermolen and Gefen (2012)
Friction coefficient	$\hat{\mu }$	g.cell.muHat	0.2	–	Vermolen and Gefen (2012)
Adhesive scaling factor	$\hat{\lambda }$	g.cell.lambda	15	–	–
Density of an EC	$P_c$	g.cell.densityC	$1.030\times 10^{-3}$	$\hbox {ng} \upmu \hbox {m}^{-3}$	Urbanchek et al. (2001)
St. dev. of stoch. movement	$\sigma _W$	g.cell.sigmaW	[0 - 0.1]	$\upmu \hbox {m}$	–
Gravitational constant	*g*	g.cell.gravitation	$9.810\times 10^{+6}$	$\upmu \hbox {m} \hbox {s}^{-2}$	–
Max. prob. stalk becoming tip	$p_{ST}$	g.cell.pMaximumS2T	$4.000\times 10^{-6}$	$\hbox {s}^{-1}$	–
Governs tip cell stimulation	$p_s$	g.cell.pStimulation	4	–	–
Governs tip cell inhibition	$p_i$	g.cell.pInhibition	10	–	–
Max. prob. tip becoming stalk	$p_{TS}$	g.cell.pMaximumT2S	$1.000\times 10^{-6}$	$\hbox {s}^{-1}$	–

**Table 5:** Substrate parameters

Parameter	Symbol	Code name	Value	Dimension	Source
Elastic modulus fibrin matrix	$E_F$	g.sub.Ef	10	$\hbox {kPa} = \hbox {nN}\, \upmu \hbox {m}^{-2}$	Rowe et al. (2007)
Elastic modulus BM	$E_B$	g.sub.Eb	20	$\hbox {kPa} = \hbox {nN}\, \upmu \hbox {m}^{-2}$	Zhu et al. (2011)
Elastic modulus ECF	$E_E$	g.sub.Ee	1	$\hbox {kPa} = \hbox {nN} \,\upmu \hbox {m}^{-2}$	–
Viscosity fibrin matrix	$\mu _F$	g.sub.viscosityF	$0.007\times 10^{+6}$	$\hbox {ng}\, \upmu \hbox {m}^{-1} \hbox {s}^{-1}$	Ehrlich et al. (1952)
Viscosity BM	$\mu _B$	g.sub.viscosityB	$0.007\times 10^{+6}$	$\hbox {ng}\, \upmu \hbox {m}^{-1} s^{-1}$	Est. based on Ehrlich et al. (1952)
Viscosity ECF	$\mu _E$	g.sub.viscosityE	$0.001\times 10^{+6}$	$\hbox {ng}\, \upmu \hbox {m}^{-1} \hbox {s}^{-1}$	Streeter et al. (1998)
Density fibrin matrix	$\rho _F$	g.sub.densityF	$1.060\times 10^{-3}$	$\hbox {ng}\, \upmu \hbox {m}^{-3}$	–
Density BM	$\rho _B$	g.sub.densityB	$1.060\times 10^{-3}$	$\hbox {ng}\, \upmu \hbox {m}^{-3}$	–
Density ECF	$\rho _E$	g.sub.densityE	$0.9933\times 10^{-3}$	$\upmu \hbox {m}$	Streeter et al. (1998)

The values for $p_m, p_s$ and $p_i$ should be found based on experimental data. Microscopic images show us that for large concentrations of VEGF ($25\,\frac{\mathrm{ng}}{\mathrm{mL}}$), we see around one sprout for every fifty ECs after two days. We assume that every sprout is led by one tip cell. Furthermore, we assume that all tip cells are selected at 12 h after stimulation with VEGF and that no additional tip cells are formed due to inhibition by the existing ones afterwards. This means that a cell has a probability of 0.02 per 12 h of becoming a tip cell not changing back again. This is equivalent to $\frac{0.02}{12\times 3600} = 4.6 \times 10^{-7}$ per second. The VEGF concentration is not maximal over the entire domain, so we choose $p_m = 4 \times 10^{-6}$. We know that the inhibiting effect of DLL4 is stronger than the stimulating effect by VEGF, so we model $p_i = 10$ and $p_s = 4$ and conclude that these values are suitable for our modelling purposes. Better estimations for these values might be obtained by fitting the model results to the experimental observations, but this is cumbersome due to the long execution time of the model. An investigation of the magnitude of these parameters reasoning from a more biochemical point of view is a useful recommendation.

### Parameters and Domain

Our model uses a number of parameters, most of a physical or chemical nature. We try to find accurate values for these parameters in the literature as much as possible although some values are hard to estimate. We list all our parameters with description, symbol, programming code name, value, dimension and where possible a source in Tables 2, 3, 4 and 5. The elastic modulus of endothelial cells $E_c$ is approximately 10 kPa according to Kuznetsova et al. (2007). They found this value in a study using atomic force microscopy probing.

According to the work of Ganz et al. (2006), traction forces $F_i$ are also higher than the 1 nN used by Vermolen and Gefen (2012). Reinhart-King et al. (2003) conducted in vitro studies measuring the forces exerted by ECs on polyacrylamide substrates. On page 1578, we see see the relation between cell area and exerted force of ECs. Since we model our cell radius as $R = 22.5\,\upmu \hbox {m}$, we have an area of $22.5^2 \pi = 1590\,\upmu \hbox {m}^2 = 1.59 \times 10^{-5}\,\hbox {cm}^2$. The graph gives a force of $0.1 \hbox {dyne} = 10^{-6} N = 1000\,\hbox {nN}$, and we choose to use this value in our model.

Elasticity of fibrin matrix $E_F$ is approximately 10 kPa according to Rowe et al. (2007). Zhu et al. (2011) propose values for the elastic modulus of collagen–chitosan scaffolds in the order of 10 kPa. Since BM consist of collagen and more stiff components, we use an estimate $E_B = 20\,\hbox {kPa}$. Plank et al. (2002) set the diffusion coefficient for VEGF $D_V = 3.6 \times 10^{-3} \frac{\hbox {mm}^2}{\hbox {h}} = 1 \frac{\upmu \hbox {m}^2}{\hbox {s}}$ (in matrigel). We have found no references on the diffusion coefficients of the concentrations DLL4, uPA and MMP so we estimate them using their molecular weights. We assume that substances diffuse more slowly for larger molecular weights. All four substances are sold commercially, and molecular weights *m* are specified very accurately. We see that $m_V \approx 38.2\,\hbox {kDa}$, $m_D \approx 75\,\hbox {kDa}$, $m_U \approx 31\,\hbox {kDa}$ and $m_M \approx 72\,\hbox {kDa}$. We estimate

$$\begin{aligned} D_D= & {} \frac{m_V}{m_D} D_V = 0.51, \quad D_U = \frac{m_V}{m_U} D_V = 1.23,\\ D_M= & {} \frac{m_V}{m_M} D_V = 0.53, \end{aligned}$$

all measured in $\frac{\upmu \hbox {m}^2}{\hbox {s}}$. Contradictorily, Bauer et al. (2009) set the diffusion coefficient for VEGF to 1698 $\frac{\upmu \hbox {m}^2}{\hbox {s}}$ and Miura and Tanaka (2009) measured in an in vitro experiments that the VEGF diffusion constant in matrigel (similar to fibrin matrix) is equal to $278\,\frac{\upmu \hbox {m}^2}{\hbox {s}}$. The coefficient used by Plank et al. (2002) seems to make the most physical sense and produces reasonable results in our computational model. Further research into the difference between these values might be useful.

Plank et al. (2002) furthermore set the VEGF uptake rate $r_V$ within cells to $8.66 \times 10^{-5} \frac{\hbox {mm}^2}{\hbox {h}} = 0.024 \frac{\upmu \hbox {m}^2}{\hbox {s}}$. Note that Plank et al. work in a 2D setting and that in the 3D setting, the dimension would be $\frac{\hbox {mm}^3}{\hbox {s}}$. We set the uptake rates $r_D$, $r_U$ and $r_M$ equal to this value since we could not find any references concerning these quantities. The degrading rate $r_F$ of the fibrin matrix fraction by uPA is, according to Lutolf et al. (2003), of magnitude 1.21 $\hbox {s}^{-1}$, and we use this value in our model. We estimate that the degrading rate $r_B$ of the BM by MMP is of the same magnitude.

Viscosity of water is $0.6531 \times 10^{-3} \frac{\mathrm{Ns}}{\mathrm{m}^2}$ at 40 degrees centigrade and $0.7978 \times 10^{-3} \frac{\mathrm{Ns}}{\mathrm{m}^2}$ at 30 degrees centigrade according to Streeter et al. (1998). Our experiment is conducted in an incubator at 37 degrees, and we therefore take $\mu _E = 0.6965 \times 10^{-3} \frac{\mathrm{Ns}}{\mathrm{m}^2}$. Viscosity fibrin matrix is approximately $7.000 \times 10^{-3} \frac{\mathrm{Ns}}{\mathrm{m}^2}$ according to Ehrlich et al. (1952). According to Urbanchek et al. (2001), the density of myocytic cells is $1.060\times 10^{-3} \frac{\mathrm{kg}}{\mathrm{cm}^3}$. Water at 37 degrees centigrade has a density of $0.9933\times 10^{-3} \frac{\mathrm{g}}{\mathrm{cm}^3}$. We model one well in a 96-well plate. We take the same dimensions of diameter 7 mm (i.e. $d_\mathrm{top} = 7000 \upmu \hbox {m}$) and a height determined by filling the well with $v_F = 100 \upmu \hbox {L}$ fibrin matrix and 100 $\upmu \hbox {L}$ fluid to a total of $v = 200\,\upmu \hbox {L}$ with $N = 20.000$ suspended EC’s. This mimics the laboratory setting one-to-one. To decrease computational time and load on memory, we introduce a linear scaling factor $\xi$, and we have scaled domain parameters $\tilde{v} = \frac{v}{\xi ^3}, \tilde{v}_F = \frac{v_F}{\xi ^3}, \tilde{d}_\mathrm{top} = \frac{d_\mathrm{top}}{\xi ^2}$ and $\tilde{N} = \frac{N}{\xi ^2}$ to keep an equal aspect ratio of the domain and a monolayer of cells that is constant in density (cells per $\upmu \hbox {m}^2$).
